# Oncogenic RTKs sensitize cancer cells to ferroptosis via c-Myc mediated upregulation of ACSL4

**DOI:** 10.1038/s41419-024-07254-9

**Published:** 2024-11-27

**Authors:** Na Sun, Jiawa Wang, Jianhua Qin, Shuang Ma, Jing Luan, Guoyuan Hou, Wei Zhang, Minghui Gao

**Affiliations:** 1 The HIT Center for Life Sciences, School of Life Science and Technology, Harbin Institute of Technology, Harbin, China; 2 Department of Microbiology and Immunology, Weill Cornell Medicine, New York, NY USA

**Keywords:** Cell death, Targeted therapies

## Abstract

Alteration or abnormal activation of RTKs have been recurrently observed and recognized as an important driving factor in the progression of many human cancers. Ferroptosis, an iron-dependent form of regulated necrosis triggered by the accumulation of lethal lipid peroxides on cell membranes, has been implicated in various tumor types. Here we reported that oncogenic RTKs/RAS/RAF/c-Myc axis promotes cancer cells to ferroptosis. Mechanistically, c-Myc binds to the promoter region of ACSL4 and promotes the expression of ACSL4, thereby sensitizes cells to ferroptosis. We further showed that RTKs/RAS/RAF promote ferroptosis by upregulating c-Myc mediated expression of ACSL4 in cancer cells. Notably, overexpression of RTKs enhances the vulnerability of melanoma to the ferroptosis inducer in mouse xenograft model. These findings may provide an attractive intervention strategy to target cancers with oncogenic activation of RTKs via a ferroptosis-inducing approach.

## Introduction

Ferroptosis, a form of cell death driven by iron-dependent lipid peroxidation, is regulated by multiple cellular metabolic processes, including redox homeostasis, iron handling, mitochondrial activity and the metabolism of amino acids, lipids and glucose [1]. Upon glutamate-cystine antiport system Xc− is inhibited and import of cystine is blocked, ferroptosis is triggered due to cysteine deprivation and glutathione depletion [2]. Glutathione peroxidase 4 (GPX4), the central negative regulator of ferroptosis [3], protects cells by catalyzing the reduction and thereby detoxifies phospholipid hydroperoxides (PLOOHs) in mammalian cells. Certain cancer types are inherently susceptible to ferroptosis owing to their unique metabolic features. In epithelial cells, the intercellular interactions and intracellular NF2–YAP signaling play a crucial role in dictating ferroptotic death [4]. In non-small cell lung cancer cells (NSCLC), the LKB1-AMPK axis negatively regulates ferroptosis by inhibiting fatty acid synthesis [5]. As such, pharmacological induction of ferroptosis holds great potential for the treatment of certain cancers.

Receptor tyrosine kinases (RTKs) are a group of transmembrane cell-surface proteins that play an important role as signal transducers [6]. They regulate essential cellular processes like proliferation, apoptosis, differentiation and metabolism [7, 8]. Alteration of RTKs occurs across a broad spectrum of cancers, emphasizing their crucial role in cancer initiation, progression and their potential as therapeutic targets [9]. Dysregulation of RTKs/RAS/RAF/c-Myc signaling leads to an assortment of human diseases, most notably, cancers [10–12]. Although the RTKs/RAS/RAF/c-Myc signaling pathway plays a role in regulating ferroptosis [13–16], the detailed mechanism by which they regulate ferroptosis remains to be further investigated.

Here we show that oncogenic activation of RTKs/RAS/RAF signaling pathway promotes cancer cell ferroptosis via c-Myc mediated upregulation of ACSL4. Moreover, we identified a new regulatory mechanism by which the proto-oncogenic transcription factor c-Myc promotes ferroptosis through upregulating the ferroptosis modulator ACSL4. ACSL4 upregulation further confers sensitivity to ferroptosis induced by cysteine deprivation and RSL3 treatment. Finally, elevated EGFR or ErbB2 levels sensitize cancer cells to ferroptosis-inducing treatment in a mouse xenograft model. Our results demonstrate the crucial role of RTKs/RAS/RAF/c-Myc signaling in dictating ferroptotic death, and suggest that malignant mutations in this signaling pathway could predict the responsiveness of cancer cells to future ferroptosis-inducing therapies.

## Results

### Oncogenic RTKs promote cancer cells ferroptosis

**Fig. 1:**
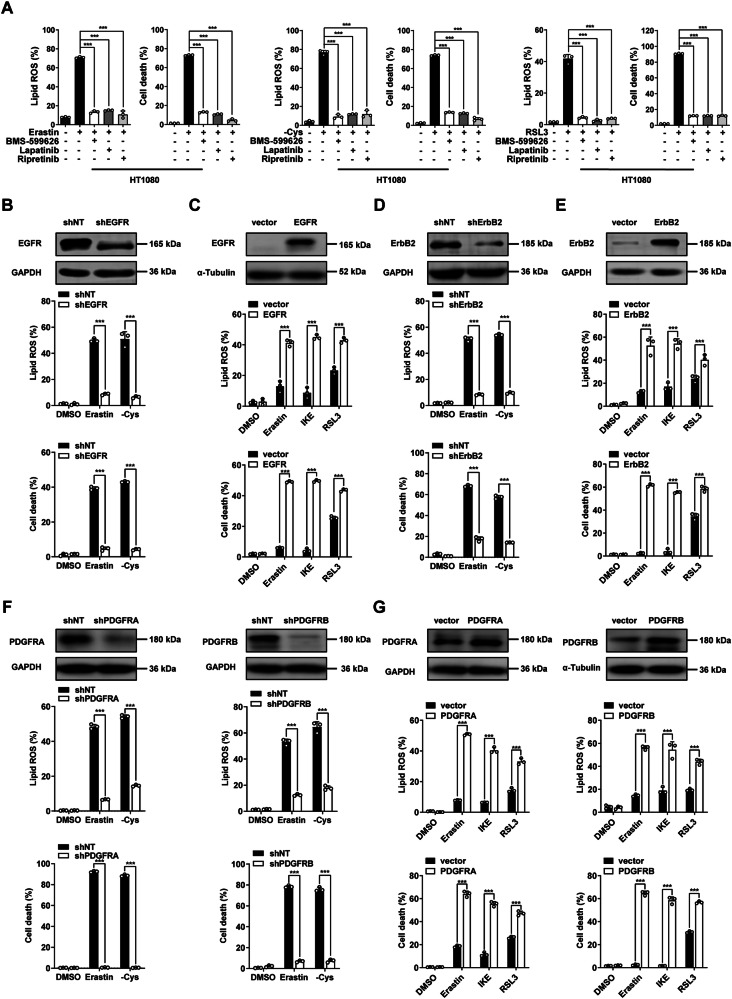
RTKs enhance the sensitivity of cancer cells to ferroptosis. **A** RTKs inhibitors block ferroptosis. HT1080 cells were treated with erastin (10 μM for 16 h), cystine deprivation (24 h), or RSL3 (0.2 μM for 5 h), in the presence of RTKs inhibitors BMS-599626 (10 μM), Lapatinib (10 μM), and Ripretinib (10 μM) or not. For cell death measurement, cells as indicated were stained with PI solution and analyzed by flow cytometry. For lipid ROS measurement, cells treated with erastin (10 μM for 14 h), cystine deprivation (20 h) or RSL3 (0.2 μM for 4 h), and lipid ROS was stained with C11-BODIPY solution and analyzed by flow cytometry. **B** Knocking down of EGFR suppresses ferroptosis in HT1080 cells. Western blot images show the expression of indicated protein. Cell death was measured as (**A**) following treatment with erastin (10 μM for 16 h) or cystine deprivation (24 h). Lipid ROS was measured as Fig. 1A following treatment with erastin (10 μM for 14 h) or cystine deprivation (20 h). **C** Overexpression of EGFR promotes ferroptosis in B16-F10 cells. Western blot images show the expression of indicated protein. Cell death was measured as (**A**) following treatment with erastin (20 μM for 24 h), IKE (20 μM for 24 h), or RSL3 (0.1 μM for 8 h). Lipid ROS was measured as (**A**) following treatment with erastin (20 μM for 20 h), IKE (20 μM for 20 h), and RSL3 (0.1 μM for 7 h). **D** Knocking down of ErbB2 suppresses ferroptosis in HT1080 cells. Western blot images show the expression of indicated protein. Cells were treated as in Fig. 1B for cell death or lipid ROS measurement. **E** Overexpression of ErbB2 promotes ferroptosis in B16-F10 cells. Western blot images show the expression of indicated protein. Cells were treated as in (**C**) for cell death or lipid ROS measurement. **F** Knocking down of PDGFRA or PDGFRB suppresses ferroptosis in B16-F10 cells. Western blot images show the expression of indicated protein. Cell death was measured as (**A**) following treatment with erastin (20 μM for 24 h) or cystine deprivation (24 h). Lipid ROS was measured as (**A**) following treatment with erastin (20 μM for 20 h) or cystine deprivation (20 h). **G** Overexpression of PDGFRA or PDGFRB promotes ferroptosis in B16-F10 cells. Western blot images show the expression of indicated protein. Cells were treated as in Fig. 1D for cell death or lipid ROS measurement. All data are mean ± SD. from n = 3 biological replicates. * *P* < 0.05, ** *P* < 0.01, *** *P* < 0.001 by two-tailed *t*-test.

To identify potent compounds which effectively modulate cancer cells ferroptosis (including both ferroptosis enhancing compounds and ferroptosis suppressing compounds), we screened a bioactive compound library with 4417 compounds using erastin-induced human fibrosarcoma HT1080 cell ferroptosis as the activity assay (Supplementary Fig. 1A). We found that several RTKs inhibitors including EGFR/ErbB2 inhibitors (BMS-599626 and Lapatinib), and the KIT/PDGFR inhibitor (Ripretinib), significantly suppress erastin-induced lipid ROS accumulation and ferroptosis in HT1080 cells (Fig. 1A). We further confirmed that these RTKs inhibitors also effectively suppress lipid ROS accumulation and ferroptosis induced by cystine deprivation or RSL3 treatment in both HT1080 (Fig. 1A) and B16-F10 mouse melanoma cells (Supplementary Fig. 1B). We next investigated the function of RTK subfamily members in ferroptosis. The epidermal growth factor receptor (EGFR), which belongs to the ErbB family of RTKs, is frequently mutated and/or overexpressed in various human cancers and is a target of multiple cancer therapies currently in clinical use [17]. As shown in Fig. 1B, knockdown of EGFR in HT1080 cells by using a specific short hairpin RNA (shRNA) blocked lipid ROS accumulation and ferroptosis. Consistently, overexpression of EGFR in B16-F10 cells sensitizes cells to lipid ROS accumulation and ferroptosis (Fig. 1C). We then assessed the function of ErbB2 in ferroptosis sensitivity of ErbB2 knockdown (KD) cells. As shown in Fig. 1D, knocking down of ErbB2 suppresses ferroptosis. Consistently, overexpression of ErbB2 enhances cancer cell sensitivity to ferroptosis (Fig. 1E). PDGFRA and PDGFRB are classical proto-oncogenes that encode receptor tyrosine kinases responding to platelet-derived growth factor (PDGF) [18]. We found that knocking down of PDGFRA or PDGFRB in B16-F10 cells individually suppresses lipid ROS accumulation and ferroptosis (Fig. 1F). Moreover, overexpression of PDGFRA or PDGFRB in B16-F10 cells individually increased lipid ROS accumulation and the sensitivity to ferroptosis (Fig. 1G). These data suggests that oncogenic RTKs, such as EGFR, ErbB2 and PDGFRs positively regulate ferroptosis.

### Oncogenic c-Myc sensitizes cancer cells to ferroptosis

**Fig. 2:**
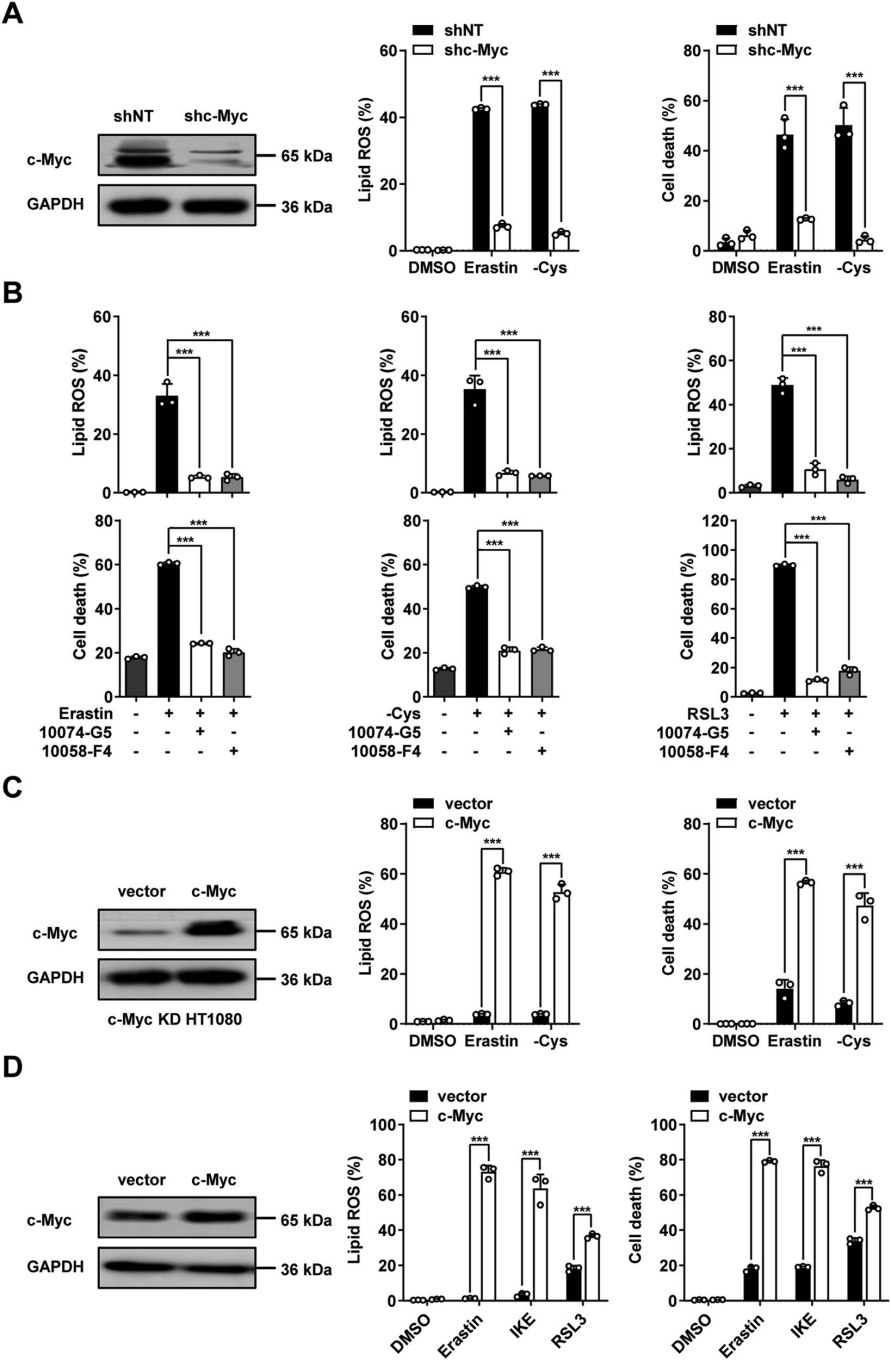
c-Myc positively regulates cancer cell ferroptosis. **A** Knocking down of c-Myc blocks ferroptosis. Western blot images show the expression of indicated protein. Cell death was measured as Fig. 1A following treatment with erastin (10 μM for 16 h) or cystine deprivation (for 24 h). Lipid ROS was measured as Fig. 1A following treatment with erastin (10 μM for 14 h) or cystine deprivation (for 20 h). **B** c-Myc inhibitors suppress ferroptosis and accumulation of lipid ROS induced by indicated ferroptosis stimuli in HT1080 cells. Erastin (10 μM), RSL3 (0.2 μM), 10074-G5 (50 μM), 10058-F4 (50 μM). **C** Reconstitution of c-Myc back to c-Myc KD HT1080 cells restores its ferroptosis sensitivity. Western blot images show the expression of indicated protein. Cell death and lipid ROS were measured as Fig. 1A. **D** Overexpression of c-Myc enhances cancer cell ferroptosis sensitivity in B16-F10 cells. Western blot images show the expression of indicated protein. Cell death was measured as Fig. 1A following treatment with erastin (20 μM for 24 h), IKE (20 μM for 24 h), or RSL3 (0.1 μM for 8 h). Lipid ROS was measured as Fig. 1A following treatment with erastin (20 μM for 20 h), IKE (20 μM for 20 h) and RSL3 (0.1 μM for 7 h). All data are mean ± SD. from n = 3 biological replicates. *P < 0.05, **P < 0.01, ***P < 0.001 by two-tailed *t*-test.

EGFR mediates the key downstream pathway MAPK cascade signaling including RAS/RAF/MEK/ERK1/2 signaling kinase, which ultimately activate downstream c-Myc [19]. The c-Myc oncogene, like its family members N-MYC and L-MYC, is a transcription factor that dimerizes with MAX to bind DNA and regulate gene expression [20, 21]. We thus investigated the function of c-Myc in ferroptosis. We observed that knocking down of c-Myc attenuates ferroptosis sensitivity in both HT1080 cells and MDA-MB-231 cells (Fig. 2A and Supplementary Fig. 2A). Consistently, pharmacological inhibition of c-Myc suppressed lipid peroxidation and ferroptosis (Fig. 2B and Supplementary Fig. 2B). Reconstitution of c-Myc back to c-Myc KD cells restored the lipid ROS accumulation and ferroptosis sensitivity (Fig. 2C and Supplementary Fig. 2C). More importantly, overexpression of c-Myc increased lipid ROS accumulation and the sensitivity to ferroptosis in B16-F10 cells (Fig. 2D). Together, these results suggest that c-Myc is a positive regulator of ferroptosis.

### c-Myc promotes ferroptosis by upregulating ACSL4

**Fig. 3:**
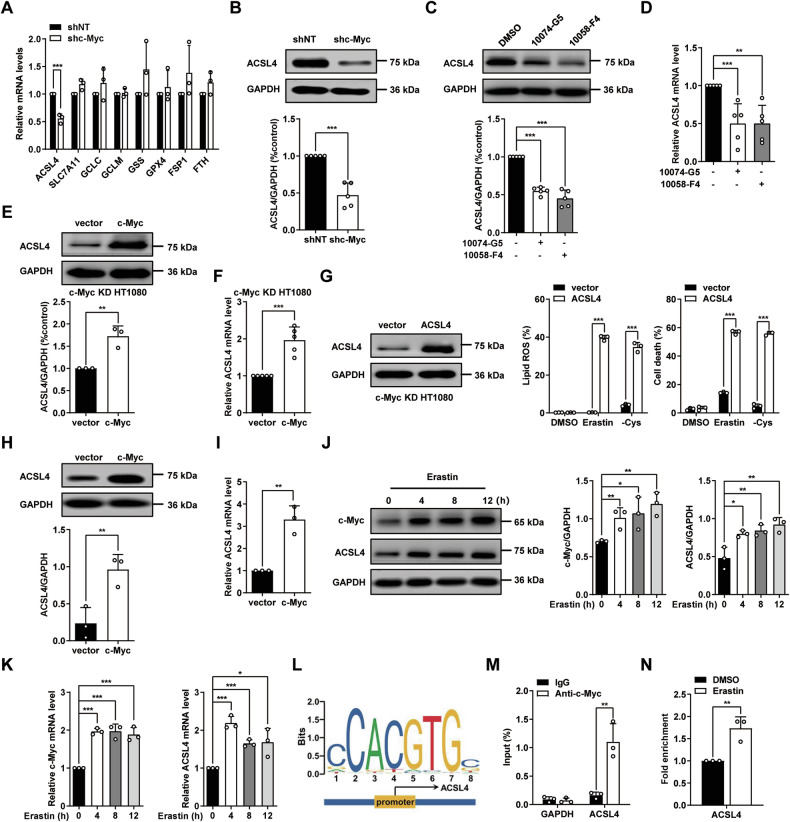
c-Myc positively regulates ferroptosis by upregulating ACSL4. **A** Knocking down of c-Myc decreases the mRNA expression of ACSL4 in HT1080 cells. RT–qPCR analyzes the expression of indicated genes. **B** Knocking down of c-Myc decreases the protein expression of ACSL4 in HT1080 cells. Western blot images show the expression of indicated protein. **C** c-Myc inhibitors suppress the protein level of ACSL4 in HT1080 cells. Cells treated with 50 μM 10074-G5 or 50 μM 10058-F4 for 24 h. n = 5. **D** c-Myc inhibitors suppress the mRNA level of ACSL4. RT–qPCR analyzes the expression of ACSL4. n = 5. **E** Reconstitution of c-Myc back to c-Myc KD HT1080 cells restores the protein level of ACSL4. Western blot images show the expression of indicated protein. **F** Reconstitution of c-Myc back to c-Myc KD HT1080 cells restores the mRNA level of ACSL4. RT–qPCR analyzes the expression of ACSL4. n = 5. **G** Overexpression of ACSL4 in c-Myc KD HT1080 cells restore ferroptosis sensitivity. Western blot images show the expression of indicated protein. Cell death was measured following treatment with erastin (10 μM for 16 h) or cystine deprivation (24 h). Lipid ROS was measured following treatment with erastin (10 μM for 14 h) or cystine deprivation (20 h). **H** Overexpression of c-Myc increases the protein level of ACSL4 in B16-F10 cells. Western blot images show the expression of indicated protein. **I** Overexpression of c-Myc increases the mRNA level of ACSL4 in B16-F10 cells. RT–qPCR analyzes the expression of ACSL4. **J** Erastin (20 μM) increases the protein level of c-Myc and ACSL4 in HT1080 cells in a time dependent. Western blot images show the expression of indicated protein. **K** Erastin (20 μM) increases the mRNA level of c-Myc and ACSL4 in HT1080 cells in a time dependent. RT–qPCR analyzes the expression of ACSL4. **L** Putative c-Myc binding site on the promoter region of ACSL4. **M** c-Myc binds to the promoter region of ACSL4 in HT1080 cells. Chromatin-IP was performed using c-Myc antibody or control IgG. Values are percentage of input. **N** Erastin promotes c-Myc binding to the promoter region of ACSL4 in HT1080 cells. Chromatin-IP was performed as Fig. 3 M. erastin: 20 μM for 6 hours. Enrichment was normalized to the IgG control. All data are mean ± SD. from n = 3 biological replicates. *P < 0.05, **P < 0.01, ***P < 0.001 by two-tailed *t*-test.

Next, we sought to determine how c-Myc regulates ferroptosis sensitivity. We examined the RNA level change of various well-studied ferroptosis regulators upon c-Myc depletion. We observed that only the expression of acyl-CoA synthetase long-chain family member 4 (ACSL4), a crucial positive regulator of ferroptosis, decreased in both c-Myc KD HT1080 cells and c-Myc KD MDA-MB-231 cells (Fig. 3A and Supplementary Fig. 3A). ACSL4 promotes ferroptosis through ligating long-chain PUFAs with coenzyme A [22, 23]. We then found the ACSL4 protein was reduced (Fig. 3B and Supplementary Fig. 3A). Consistently, two c-Myc specific inhibitors significantly decrease ACSL4 expression on both protein and RNA level (Fig. 3C, D and Supplementary Fig. 3B). Reconstitution of c-Myc back to c-Myc KD cells could restore the expression of ACSL4 both on protein level and RNA level (Fig. 3E, F and Supplementary Fig. 3C). Moreover, overexpression of ACSL4 in c-Myc KD HT1080 cells could rescue lipid peroxidation and their sensitivity to ferroptosis (Fig. 3G). Furthermore, overexpression of c-Myc increases the expression of ACSL4 in B16-F10 cells on both protein level (Fig. 3H) and RNA level (Fig. 3I). Importantly, we observed that ferroptosis stimuli induces significantly upregulation of c-Myc and ACSL4 both on protein level (Fig. 3J and Supplementary Fig. 3D) and RNA level (Fig. 3K and Supplementary Fig. 3E). As shown in Fig. 3L, there are four putative c-Myc binding sites in the promoter region of ACSL4 according to the JASPAR transcription factor binding profile database [24]. We then confirmed that c-Myc binds to the -979 site in the promoter region of ACSL4 by chromatin IP assay (Fig. 3M). More importantly, erastin could promote c-Myc binding to the promoter region of ACSL4 (Fig. 3N). These data suggest that c-Myc positively regulates ferroptosis by directly upregulating the expression of ACSL4.

### Oncogenic RTKs promote ferroptosis via c-Myc mediated upregulation of ACSL4

**Fig. 4:**
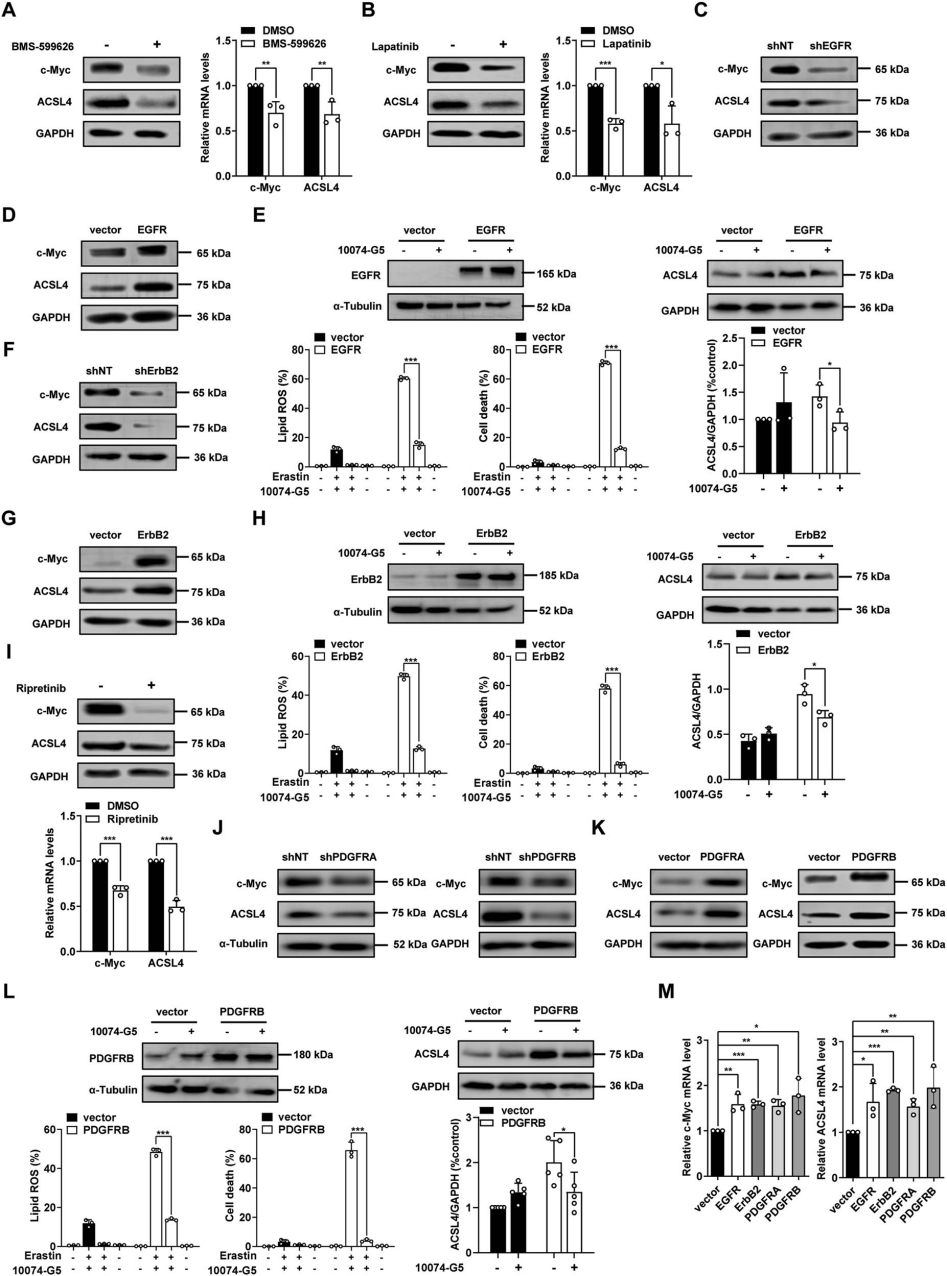
RTKs promote ferroptosis by upregulating c-Myc and ACSL4. **A**, **B** EGFR and ErbB2 inhibitors BMS-599626 (20 μM for 24 h) and Lapatinib (20 μM for 24 h) suppress the expression of c-Myc and ACSL4 on both protein level and RNA level in HT1080 cells. Western blot images show the expression of indicated protein. RT–qPCR analyzes the expression of c-Myc and ACSL4. **C** Knocking down of EGFR suppresses the expression of c-Myc and ACSL4 in HT1080 cells. Western blot images show the expression of indicated protein. **D** Overexpression of EGFR increases the expression of c-Myc and ACSL4 in B16-F10 cells. Western blot images show the expression of indicated protein. **E** c-Myc inhibitor 10074-G5 (50 μM for 24 h) blocks elevated ferroptosis sensitivity and ACSL4 expression induced by EGFR overexpression in B16-F10 cells. **F** Knocking down of ErbB2 suppresses the expression of c-Myc and ACSL4 in HT1080 cells. Western blot images show the expression of indicated protein. **G** Overexpression of ErbB2 increases the expression of c-Myc and ACSL4 in B16-F10 cells. Western blot images show the expression of indicated protein. **H** c-Myc inhibitor 10074-G5 (50 μM for 24 h) blocks elevated ferroptosis sensitivity and ACSL4 expression induced by ErbB2 overexpression in B16-F10 cells. **I** PDGFR inhibitor Ripretinib (20 μM for 24 h) suppresses the expression of c-Myc and ACSL4 on both protein level and RNA level in HT1080 cells. Western blot images show the expression of indicated protein. RT–qPCR analyzes the expression of c-Myc and ACSL4. **J** Knocking down of PDGFRA or PDGFRB suppresses the expression of c-Myc and ACSL4 in B16-F10 cells. Western blot images show the expression of indicated protein. **K** Overexpression of PDGFRA or PDGFRB increases the expression of c-Myc and ACSL4 in B16-F10 cells. Western blot images show the expression of indicated protein. **L** c-Myc inhibitor 10074-G5 (50 μM for 24 h) blocks elevated ferroptosis sensitivity and ACSL4 expression induced by PDGFRB overexpression in B16-F10 cells. **M** Overexpression of RTKs increases the mRNA level of c-Myc and ACSL4 in B16-F10 cells. RT–qPCR analyzes the expression of c-Myc and ACSL4. All data are mean ± SD. from n = 3 biological replicates. *P < 0.05, **P < 0.01, ***P < 0.001 by two-tailed *t*-test.

Given the crucial positive role of c-Myc and ACSL4 in ferroptosis, we next investigated whether RTKs promote ferroptosis by upregulating the expression of c-Myc and ACSL4. We observed that treatment with EGFR inhibitor or ErbB2 inhibitor remarkably suppressed the expression of c-Myc and ACSL4 both on protein level and RNA level in HT1080 (Fig. 4A, B) and B16-F10 cells (Supplementary Fig. 4A, B). Similarly, knocking down of EGFR significantly reduces the expression of c-Myc and ACSL4 in HT1080 cells (Fig. 4C). Consistently, overexpression of EGFR significantly increased the expression of c-Myc and ACSL4 in B16-F10 cells on both protein level (Fig. 4D) and RNA level (Fig. 4M). We further found that c-Myc inhibitor 10074-G5 effectively reduces the higher ferroptosis sensitivity and higher expression of ACSL4 in EGFR overexpressed B16-F10 cells (Fig. 4E). Similar to EGFR, knocking down of ErbB2 significantly reduces the expression of c-Myc and ACSL4 in HT1080 cells (Fig. 4F). Overexpression of ErbB2 significantly increased the expression of c-Myc and ACSL4 in B16-F10 cells on both protein level (Fig. 4G) and RNA level (Fig. 4M). c-Myc inhibitor 10074-G5 effectively reduces the higher ferroptosis sensitivity and higher expression of ACSL4 in ErbB2 overexpressed B16-F10 cells (Fig. 4H). Similar to the inhibitors of EGFR and ErbB2, treatment with PDGFR inhibitor significantly decreases the expression of c-Myc and ACSL4 (Fig. 4I and Supplementary Fig. 4C). Knocking down of PDGFRA or PDGFRB significantly reduces the expression of c-Myc and ACSL4 (Fig. 4J). Consistently, overexpression of PDGFRA or PDGFRB increased the expression of c-Myc and ACSL4 both on protein level (Fig. 4K) and RNA level in B16-F10 cells (Fig. 4M). Treatment with c-Myc inhibitor 10074-G5 also reduces the higher ferroptosis sensitivity and higher ACSL4 expression in PDGFRB overexpressed B16-F10 cells (Fig. 4L). Collectively, these results indicate that RTKs promote ferroptosis by upregulating c-Myc mediated expression of ACSL4 in cancer cells.

### RAS/RAF enhances ferroptosis sensitivity through upregulation of c-Myc mediated ACSL4 expression

**Fig. 5:**
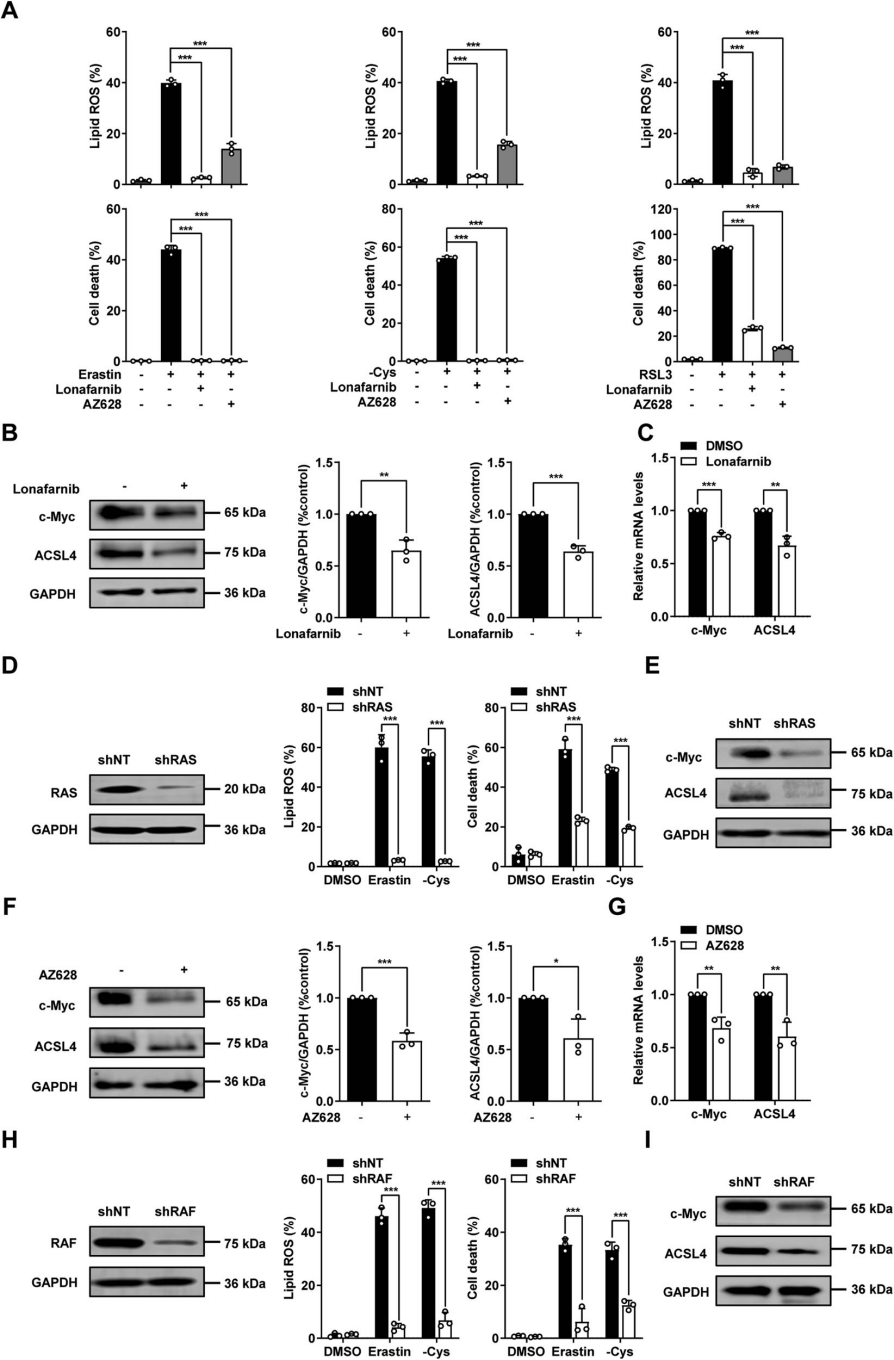
RAS/RAF enhances the sensitivity of cancer cells to ferroptosis by upregulating c-Myc and ACSL4. **A** RAS inhibitor (Lonafarnib: 10 μM) or RAF inhibitor (AZ628: 10 μM) blocks ferroptosis induced by erastin (10 μM for 16 h), cystine deprivation (24 h) or RSL3 (0.2 μM for 5 h) in HT1080 cells. Cell death and lipid ROS were measured as Fig. 1A. **B** RAS inhibitor lonafarnib (10 μM) suppresses the protein level of c-Myc and ACSL4. Western blot images and quantitative bar graphs show the expression of indicated protein. **C** RAS inhibitor lonafarnib (10 μM) suppresses the mRNA level of c-Myc and ACSL4 in HT1080 cells. RT–qPCR analyzes the expression of c-Myc and ACSL4. **D** Knocking down of RAS inhibits ferroptosis in HT1080 cells. Western blot images show the expression of indicated protein. Cell death was measured as Fig. 1A following treatment with erastin (10 μM 16 h) or cystine deprivation (24 h). Lipid ROS was measured as Fig. 1A following treatment with erastin (10 μM 14 h) or cystine deprivation (20 h). **E** Knocking down of RAS suppresses the protein level of c-Myc and ACSL4 in HT1080 cells. Western blot images show the expression of indicated protein. **F** RAF inhibitor AZ628 (10 μM) suppresses the protein level of c-Myc and ACSL4 in HT1080 cells. Western blot images and quantitative bar graphs show the expression of indicated protein. **G** RAS inhibitor lonafarnib (10 μM) suppresses the mRNA level of c-Myc and ACSL4 in HT1080 cells. RT–qPCR analyzes the expression of c-Myc and ACSL4. **H** Knocking down of RAF inhibits ferroptosis in HT1080 cells. Western blot images show the expression of indicated protein. Cell death was measured as Fig. 1A following treatment with erastin (10 μM 16 h) or cystine deprivation (24 h). Lipid ROS was measured as Fig. 1A following treatment with erastin (10 μM 14 h) or cystine deprivation (20 h). **I** Knocking down of RAS suppresses the protein level of c-Myc and ACSL4 in HT1080 cells. Western blot images show the expression of indicated protein. All data are mean ± SD. from n = 3 biological replicates. *P < 0.05, **P < 0.01, ***P < 0.001 by two-tailed *t*-test.

RAS encodes a GTPase that relays signals from growth factor receptors to downstream signaling cascades [25]. RAF family members act directly downstream of RAS, they play an important role in oncogenesis [26]. In 2012, Dixon et al. reported that the small molecule erastin triggered ferroptosis in oncogenic RAS overexpressed cells [13]. However, how oncogenic RAS overexpression sensitizes cancer cells to ferroptosis remains unclear. To address this question, we examined whether the function of RAS/RAF in ferroptosis depends on the regulation of c-Myc and ACSL4. Treatment with RAS inhibitor Lonafarnib or RAF inhibitor AZ628 significantly inhibited ferroptosis in HT1080 cells (Fig. 5A), MDA-MB-231 cells and B16-F10 cells (Supplementary Fig. 5A). RAS inhibitor Lonafarnib treatment significantly reduces the expression of c-Myc and ACSL4 on both protein level (Fig. 5B and Supplementary 5B) and RNA level (Fig. 5C and Supplementary 5B) in multiple cell lines. Knocking down of RAS significantly suppresses lipid ROS accumulation and ferroptosis (Fig. 5D) and reduces the expression of c-Myc and ACSL4 in HT1080 cells (Fig. 5E). Similarly, AZ628 treatment reduced c-Myc and ACSL4 expression at both the protein level (Fig. 5F and Supplementary 5C) and RNA levels (Fig. 5G and Supplementary 5C) in multiple cell lines. Consistently, knocking down of RAF in HT1080 cells significantly suppressed lipid ROS accumulation and ferroptosis (Fig.5H) and reduced the expression of c-Myc and ACSL4 (Fig. 5I). Taken together, these results indicate that oncogenic RAS/RAF promotes ferroptosis by upregulating c-Myc mediated ACSL4 expression.

### RTKs promote cancer cells sensitivity to ferroptosis in vivo

**Fig. 6:**
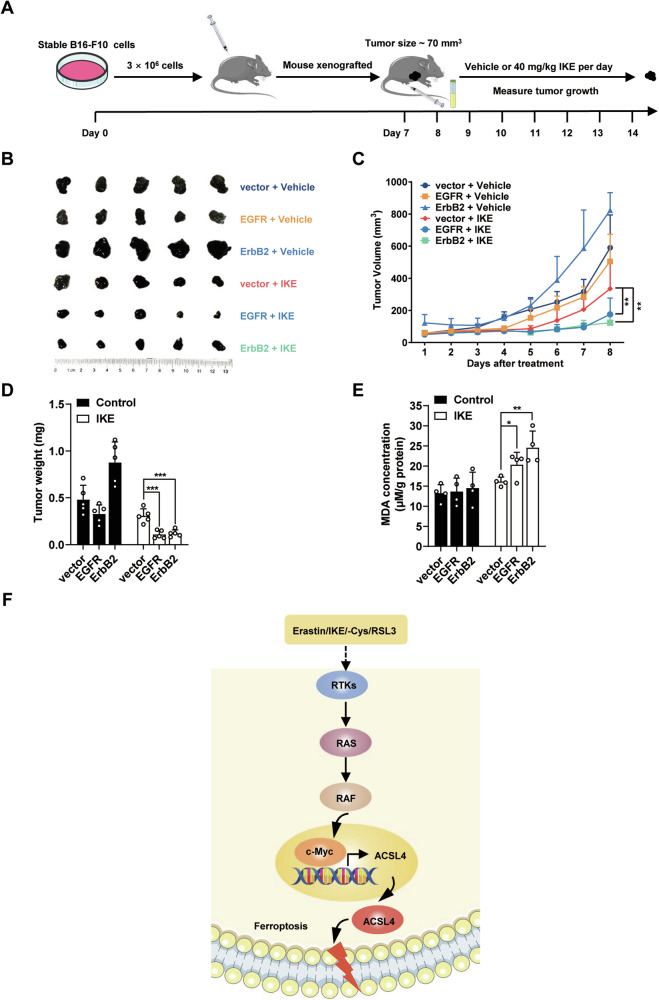
RTKs promote cancer cells sensitivity to ferroptosis in vivo. **A** Schematic illustration of the experimental design of mouse xenograft model. **B** High level of RTKs sensitize cancer cells to ferroptosis-inducing treatment. Representative photographs of tumors in the vehicle group and IKE group. The indicated B16-F10 cells were injected subcutaneously into C57BL/6 mice. **C** Growth curves of tumors after drug treatment. **D** Tumor weight after drug treatment. **E** High level of RTKs increases MDA level of cancer cells upon ferroptosis-inducing treatment. **F** Working model. RTKs/RAS/RAF/c-Myc axis is activated during ferroptosis, which results in upregulation of ACSL4 expression and enhancing the sensitivity of cancer cells to ferroptosis. All data are mean ± SD. n = 5. *P < 0.05, **P < 0.01, ***P < 0.001 by two-tailed *t*-test.

Since oncogenic activation or overexpression of RTKs frequently occur in many different cancers and play a crucial role in cancer development [27], we examined whether RTKs status could be a biomarker for future ferroptosis-inducing cancer treatment using mouse xenograft model. The B16-F10 mouse xenograft model was performed as shown in Fig. 6A. Imidazole ketone erastin (IKE), a more potent and metabolically stable generation of erastin [28], was used in our mouse xenograft model. We observed that high expression of RTKs including EGFR and ErbB2 sensitized cancers to ferroptosis-inducing tumor treatment (Fig. 6B–D). MDA is one of the most frequently measured biomarkers of lipid peroxidation in ferroptosis [29]. We further observed a significantly higher MDA level in EGFR or ErbB2 overexpressed tumor tissues than vector control tumor tissue upon IKE treatment (Fig. 6E), suggesting more ferroptosis is induced by IKE treatment in EGFR or ErbB2 overexpressed tumor tissues. These findings suggest that EGFR and ErbB2 status might be a valuable biomarker for future ferroptosis-inducing cancer therapy in certain cancer patients.

## Discussion

Collectively, in this study we demonstrated that oncogenic RTKs/RAS/RAF/c-Myc signaling axis promotes ferroptosis. Oncogene activation can render cancer cells susceptible to ferroptosis [30]. Poursaitidis et al. showed that EGFR and BRAF mutant cells are sensitive to ferroptosis and sensitivity to depletion of cystine was related to activation of MAPK [15]. MYCN induces massive lipid peroxidation and sensitizes cells to ferroptosis upon cysteine depletion [31]. Ferroptosis inducers including erastin and RSL3 were identified from a screening that specifically killed oncogenic RAS overexpressed cells [32], indicating oncogenic RAS is required for ferroptosis. The Stockwell group further showed that RAS positively regulates ferroptosis through downstream MEK/ERK signaling [33]. However, it was shown that a selective and potent MEK1/2 inhibitor, PD0325901, could not block erastin or cystine starvation induced ferroptosis in MEFs, suggesting MEK/ERK signaling may not be essential for ferroptosis [34]. Here, we found ferroptosis inducers treatment leads to activation of RTKs/c-Myc axis, which significantly promotes lipid peroxidation and ferroptosis. Mechanistically, ferroptosis stimuli increase the expression of ACSL4 which sensitizes cells to ferroptosis, and the upregulation of ACSL4 is directly regulated by transcription factor c-Myc. The positive role of RAS in ferroptosis is consistently confirmed by independent groups. But how RAS positively regulates ferroptosis may be cell context dependent. Previous studies have demonstrated that MYCN influences multiple ferroptosis-related pathways, including transsulfuration genes [31] and TFRC [14]. We also found that c-Myc negatively regulates the expression of GOT1 and NRF2, two negative regulators of ferroptosis (Supplementary Figure 3F–H), suggesting that c-Myc may exert broader control beyond ACSL4. Further studies should be performed to systemically investigate additional pathways which may be involved in c-Myc mediated regulation of ferroptosis.

As the RTKs/RAS/RAF/c-Myc signaling axis is frequently overactivated in cancer, this study has clear implications for cancer therapies—malignant alterations of several components in this signaling axis sensitize cancer cells to ferroptosis. Here we found that growth of tumor tissues with high expression of RTKs is significantly inhibited by ferroptosis inducer IKE in mouse xenograft model. Our finding suggests that there might be a dose-responsive window for cancers that contain certain genetic signatures and that ferroptosis-inducing cancer therapies such as IKE might have considerable benefits for these patients.

## Materials and methods

### Reagents and antibodies

Primary antibodies used in the study are anti-c-Myc (Proteintech, Cat# 10828-1-AP), anti-c-Myc (CST, Cat# 9402S), anti-ACSL4 (Santa Cruz, Cat# sc-365230), anti-EGFR (Proteintech, Cat# 66455-1-Ig), anti-ErbB2 (Proteintech, Cat# 18299-1-AP), anti-PDGFRB (Proteintech, Cat# 13449-1-AP), anti-RAS (Proteintech, Cat# 60309-1-Ig), anti-RAF (Proteintech, Cat# 66592-1-Ig), anti-GAPDH (Proteintech, Cat# 60004-1-Ig), anti-Alpha Tubulin (Proteintech, Cat# 11224-1-AP), anti-PDGFRA (HuaBio, Cat# ET1702-49), and anti-NRF2 (Proteintech, Cat# 16396-1-AP). Chemical compounds used in the study are Erastin (Selleck, Cat# S7242), Imidazole ketone erastin (IKE) (Selleck, Cat# S8877), RSL3 (Selleck, Cat# S8155), BMS-599626 (MCE, Cat# HY-10251), Lapatinib (MCE, Cat# HY-50898), Ripretinib (MCE, Cat# HY-112306), Lonafarnib (MCE, Cat# HY-15136), AZ628(MCE, Cat# HY-11004), 10074-G5(Selleck, Cat# S8426), and 10058-F4 (ApexBio, Cat# A1169).

### Cell culture

HT1080 cells, MDA-MB-231 cells, and B16-F10 cells were cultured in DMEM with high glucose, sodium pyruvate (1 mM), glutamine (4 mM), penicillin (100 U/ml), streptomycin (0.1 mg/ml) and 10% (v/v) FBS at 37 °C and 5% CO_2_.

### Induction of ferroptosis

To induce ferroptosis, cells were seeded in 12-well plates at a density of 2 × 10^5^ cells per well. For cystine-deprivation experiments, cells were washed twice with PBS and then cultured in cystine-free medium with penicillin (100 U/ml), streptomycin (0.1 mg/ml) and 10% (v/v) FBS for the indicated time. Compounds erastin, IKE, and RSL3 were also used as ferroptosis inducers as indicated.

### Bioactive Compound Library Screening

The Bioactive Compound Library (ApexBio, Cat# A1169) consisting of 4417 bioactive compounds in 96-well plates with a concentration of 10 mM in DMSO were used for the screening. HT1080 cells seeded in 24-well plates with a density of 8 × 10^4^ cells per well were treated with erastin (10 μM) plus individual compound (10 μM) from the library for 18 h. After drugs treatment, cells were stained with 100 ng/ml PI and then photographed by fluorescence microscopy or analyzed by flow cytometry.

### Measurement of cell death and lipid peroxidation

For cell death measurement:

Cells treated as indicated were stained with 100 ng/ml propidium iodide (Thermo Fisher, Cat# P1304MP) and then photographed using fluorescence microscopy or analyzed by flow cytometry.

For lipid peroxidation measurement:

Cells treated as indicated were stained with 5 μM BODIPY-C11 (Thermo Fisher, Cat# D3861) for 30 min at 37°C, and then analyzed by flow cytometry.

### Generation of stable cells

Lentiviral shRNA clones targeting human EGFR, ErbB2, PDGFRA, PDGFRB, RAS, RAF, c-Myc, and non-targeting control construct were purchased from Sigma-Aldrich. The TRC number of shRNA are listed as following: c-Myc: TRCN0000174055, RAS: TRCN0000033256, RAF: TRCN0000001064, EGFR: TRCN0000010329, ErbB2: TRCN0000039881, PDGFRA: TRCN0000321928, PDGFRB: TRCN0000321931. Plasmids pLV3-CMV-EGFR-CopGFP, pLV3-CMV-PDGFRB-CopGFP, pLV3-CMV-PDGFRA-CopGFP, pBABE-ErbB2, pBABE-c-Myc, and pIC112-ACSL4 were obtained from MiaoLing Plasmid Platform or Addgene Plasmid Platform. Retrovirus or lentivirus was packaged in 293T cells and used to infect target cells, which were then selected with puromycin for at least 3 days prior to use in experiments.

### RT–qPCR

Total RNA was extracted using TRIzol reagent (Thermo Fisher, Cat# 15596026) according to the manufacturer’s instructions. cDNA was synthesized using the cDNA Synthesis Kit (Takara, Cat# RR047A) according to the manufacturer’s instructions. RT–qPCR was performed with ChamQ Universal SYBR qPCR Master Mix (Vazyme, Cat# Q711-02) in a Real-Time PCR system (Applied Biosystems). Primer sequences are listed in the Supplementary Table.

### Chromatin-IP (CHIP) assay

HT1080 cells were crosslinked in 1% formaldehyde for 10 min, and glycine was added to a final concentration of 125 mM for 5 min. After washing with PBS twice, cells were collected in lysis buffer and sonicated using an ultrasonic homogenizer for 5 min at 35% power on ice to shear DNA to an average fragment size of 200–1000 bp. Fifty microliters of each sonicated sample were used to determine the DNA concentration and fragment size. Cell lysates were incubated with 50 μl Pierce Protein A/G Agarose (Thermo Fisher, Cat# 20421) and 10 μl ChIP-grade c-Myc antibody at 4 °C overnight. Beads were collected, washed, and treated with proteinase K for 2 h at 60 °C and RNase for 1 h at 37 °C. DNA was purified using a PCR purification kit (Thermo Fisher, Cat# K0702). DNA fragments were assessed by qPCR using the primer sequences listed in the Supplementary Table. Samples were normalized to input DNA.

### In vivo mouse xenograft model

EGFR, ErbB2, and vector overexpressed B16-F10 cells were trypsinized and counted. Six- to eight-week-old male C57BL/6 J mice were purchased from Liaoning Changsheng Biotechnology. For subcutaneous tumor models, mice were injected in the right flank with 3 × 10^6^ cells suspended in 100 μl PBS. Tumors were measured with a caliper every day. When tumors reached a mean volume of 70 mm^3^, mice with similarly sized tumors were grouped into six treatment groups and treated with vehicle (5% DMSO, 5% Tween-80, 40% PEG-300, 50% double-distilled water) or 40 mg/kg IKE (5% DMSO, 5% Tween-80, 40% PEG-300, 50% double-distilled water) via intraperitoneal injection once a day. After eight days, the mice were euthanized, and the tumors were weighed and photographed. Tumor tissues were collected for MDA assay. The study complies with all relevant ethical regulations for animal experiments.

### Tumor MDA assay

High intracellular malondialdehyde (MDA) levels indicate lipid peroxidation. The intracellular MDA concentration in tumor tissue was assessed using the MDA Assay Kit (Beyotime, Cat# S0131S) according to the manufacturer’s instructions.

### Statistical analysis

All statistical analyzes were performed using GraphPad Prism 8.0 software. Data are presented as mean ± SD. *P* values were determined by Student’s two-tailed *t*-test. Significance was set at *p* < 0.05. All data are representative of at least three independent experiments.

## Supplementary information

Primer sequences for qPCR

Supplementary information

Original full length Western blots

## Data Availability

The data that support the findings of this study are available from the corresponding author upon reasonable request.
