# Supplementary material for: Oncogenic RTKs sensitize cancer cells to ferroptosis via c-Myc mediated upregulation of ACSL4
Source: Cell Death Dis. 2024 Nov 27;15(11):861. doi: 10.1038/s41419-024-07254-9 (PMC11603294; doi:10.1038/s41419-024-07254-9)
Supplement: Supplementary file 1 — Primer sequences for qPCR [file 41419_2024_7254_MOESM1_ESM.docx]

Table S1 Primer sequences for qPCR.

| Gene | Source | Sense（5’→3’） | Antisense（5’→3’） |
| --- | --- | --- | --- |
| ACSL4 | human | CATCCCTGGAGCAGATACTCT | TCACTTAGGATTTCCCTGGTCC |
| β-ACTIN | human | CATGTACGTTGCTATCCAGGC | CTCCTTAATGTCACGCACGAT |
| c-Myc | human | CACAGCAAACCTCCTCACAG | TGCGTAGTTGTGCTGATGTG |
| FSP1 | human | GCGTTTGAGAGCAGACTAGC | CACAGTCACCAATGGCGTAG |
| SLC7A11 | human | ATGCAGTGGCAGTGACCTTT | GGCAACAAAGATCGGAACTG |
| GCLC | human | GGAGGAAACCAAGCGCCAT | CTTGACGGCGTGGTAGATGT |
| GCLM | human | TGTCTTGGAATGCACTGTATCTC | CCCAGTAAGGCTGTAAATGCTC |
| GSS | human | GGGAGCCTCTTGCAGGATAAA | GAATGGGGCATAGCTCACCAC |
| GPX4 | human | GAGGCAAGACCGAAGTAAACTAC | CCGAACTGGTTACACGGGAA |
| FTH | human | GCCAGAACTACCACCAGGAC | TGTGCAGTTCCAGTAGTGAC |
| ACSL4-Chip | human | GGAAGCCCGTTCGGCTCCGC | CGCGGTGGTGTGGCAGAGCCA |
| GAPDH-Chip | human | GGAACATGCTGAGAAACTGATGAA | CATCACAGTCTGGTTTCTTGATATCC |
| β-ACTIN | Mouse | ATTGGCAACGAGCGGTTCC | AGCACTGTGTTGGCATAGAGG |
| c-Myc | Mouse | TGGAACGTCAGAGGAGGAAC | TGTGCTCGTCTGCTTGAATG |
| ACSL4 | Mouse | CTTTGGAAGTGGCCTCACTG | AAAGCAAGTCTGTGCTGCAA |
| GOT1 | Mouse | GCGCCTCCATCAGTCTTTG | ATTCATCTGTGCGGTACGCTC |
