# Supplementary material for: Oncogenic RTKs sensitize cancer cells to ferroptosis via c-Myc mediated upregulation of ACSL4
Source: Cell Death Dis. 2024 Nov 27;15(11):861. doi: 10.1038/s41419-024-07254-9 (PMC11603294; doi:10.1038/s41419-024-07254-9)
Supplement: Supplementary file 2 — Supplementary information [file 41419_2024_7254_MOESM2_ESM.docx]

# Oncogenic RTKs sensitize cancer cells to ferroptosis via c-Myc mediated upregulation of ACSL4

## Na Sun^1^, Jiawa Wang^1^, Jianhua Qin^1^, Shuang Ma^1^, Jing Luan^1^, Guoyuan Hou^1^, Wei Zhang^2*^ and Minghui Gao^1*^

**Files included in the supplementary information:**

**Supplementary figure 1** **（Supplemental to Figure. 1）**

**Supplementary figure 2** **（Supplemental to Figure 2）**

**Supplementary figure 3** **（Supplemental to Figure 3）**

**Supplementary figure 4** **（Supplemental to Figure 4）**

**Supplementary figure 5** **（Supplemental to Figure 5）**


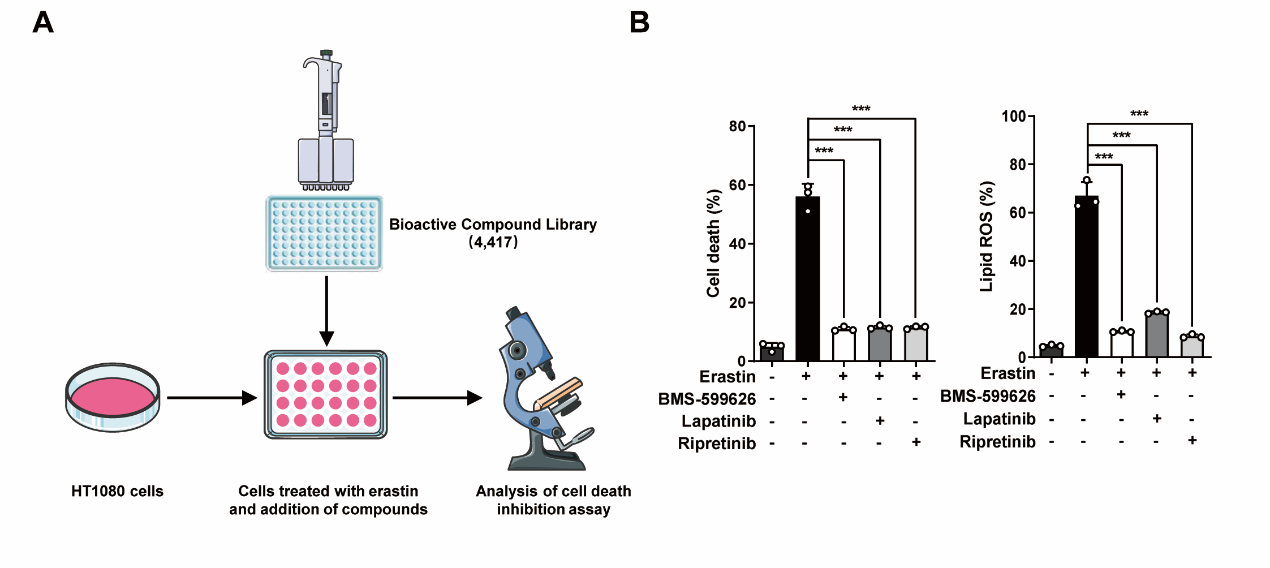


**Supplementary figure 1. RTKs enhance the sensitivity of cancer cells to ferroptosis.**

**Fig.S1 A** Overview of the ferroptosis inhibitor screening in HT1080 cells**.** Cells seeded in 24-well plates overnight were treated with erastin (10 μM) and individual small compound from the library for 16 h. Cell death was analyzed by PI staining followed by microscopy or flow cytometry. **B** RTKs inhibitors suppress erastin-induced ferroptosis in B16-F10 cells. B16-F10 Cells were treated as indicated for 24 h to measure cell death. B16-F10 cells were treated as indicated for 20 h to determine lipid ROS. Erastin (20 μM), BMS-599626 (10 μM), Lapatinib (10 μM), Ripretinib (10 μM). All data are mean ± SD. from n = 3 biological replicates. **P* < 0.05, ***P* < 0.01, ****P* < 0.001 by two-tailed *t*-test.


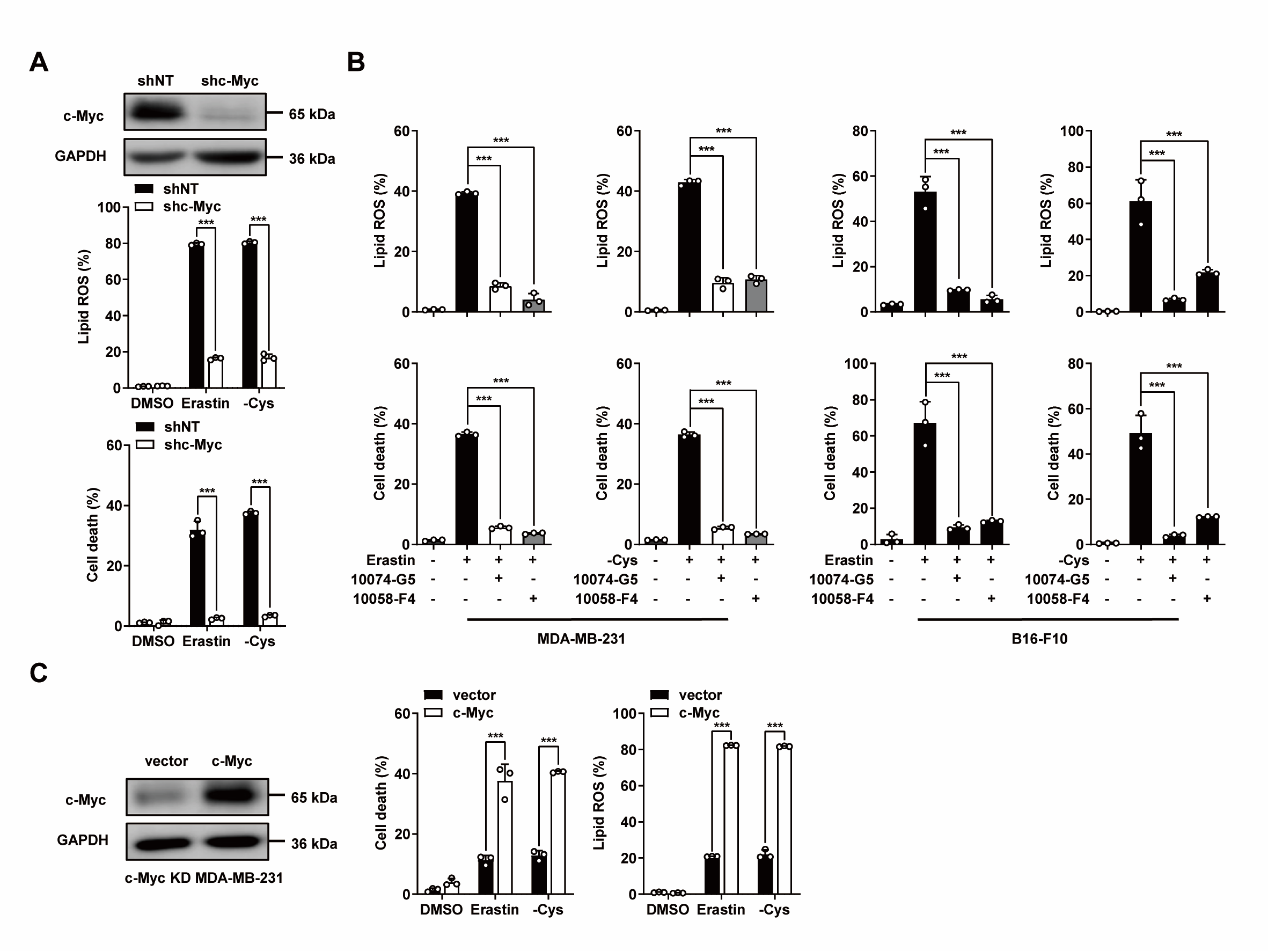


**Supplementary figure 2. c-Myc positively regulates cancer cell ferroptosis.**

**Fig.S2 A** Knocking down of c-Myc attenuates ferroptosis sensitivity in MDA-MB-231 cells. Western blot images show the expression of indicated protein. Cells as indicated were treated with erastin (10 μM for 18 h) or cystine deprivation (24 h). Cell death and lipid ROS were measured as Figure 1A. **B** c-Myc inhibitors suppress the accumulation of lipid ROS and ferroptosis induced by erastin or cystine deprivation in MDA-MB-231 and B16-F10 cells. Cell death and lipid ROS were measured as in Figure 1A. Erastin: 10 μM for MDA-MB-231 cells and 20 μM for B16-F10 cells; 10074-G5: 50 μM; 10058-F4: 50 μM. **C** Reconstitution of c-Myc back to c-Myc KD MDA-MB-231 cells restores its ferroptosis sensitivity. Western blot images show the expression of indicated protein. Cell death and lipid ROS were measured as Figure 1A. All data are mean ± SD. from n = 3 biological replicates. **P* < 0.05, ***P* < 0.01, ****P* < 0.001 by two-tailed *t*-test.


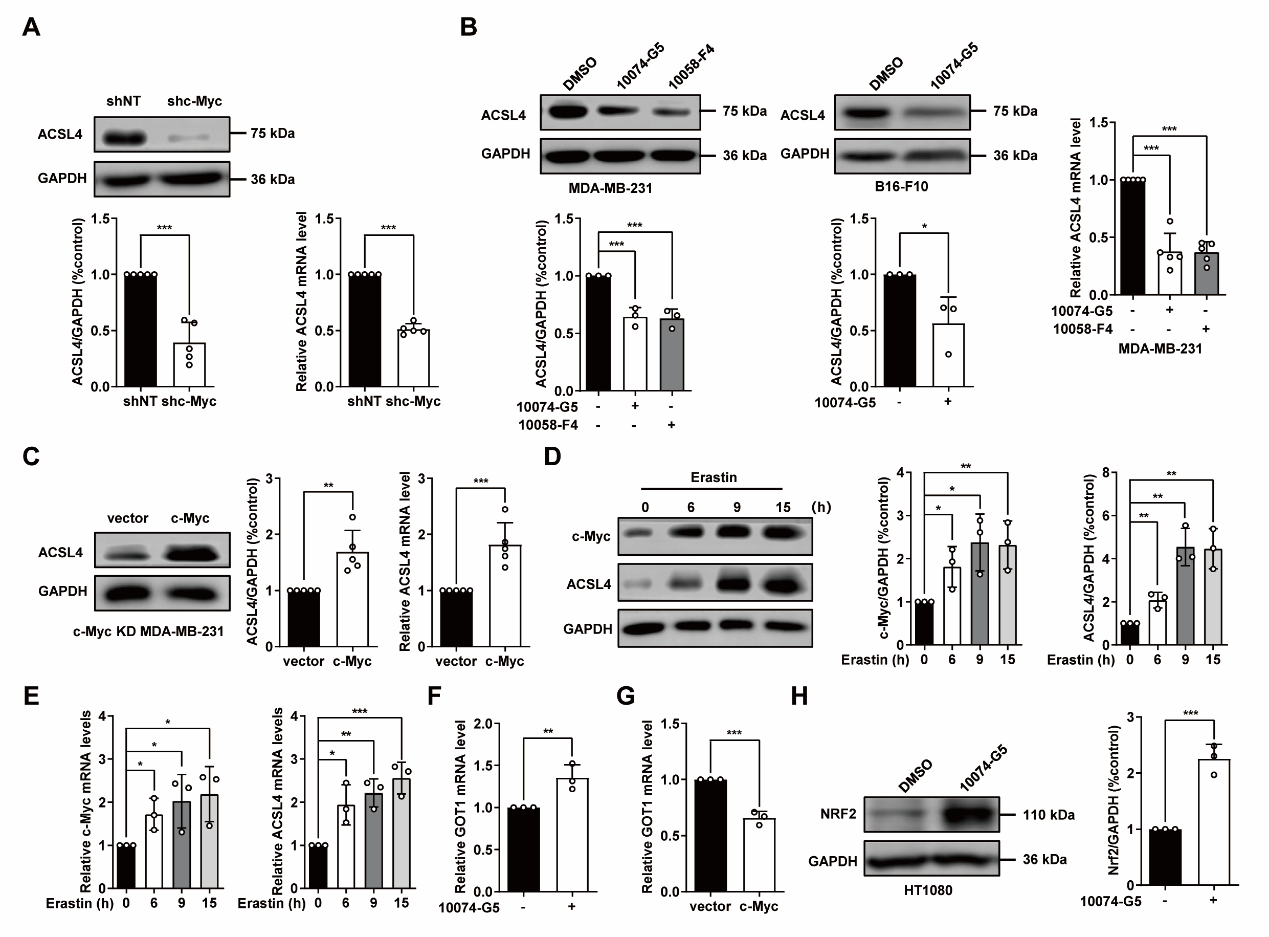


**Supplementary figure 3. c-Myc positively regulates ferroptosis by up-regulating ACSL4.**

**Fig.S3 A** Knocking down of cMyc suppresses the expression of ACSL4 on both protein level and RNA level in MDA-MB-231 cells. Western blot images show the expression of indicated protein. RT-qPCR analyzes the expression of ACSL4. n = 5. **B** c-Myc inhibitors suppress the expression of ACSL4 on both protein level and RNA level in cells as indicated. Western blot images show the expression of indicated protein. RT-qPCR analyzes the expression of ACSL4 in MDA-MB-231 cells. 10074-G5: 50 μM, 10058-F4: 50 μM, n =3. **C** Reconstituting c-Myc back to c-Myc KD MDA-MB-231 cells restores the expression of ACSL4 on both protein level and RNA level. Western blot images show the expression of indicated protein. RT-qPCR analyzes the expression of ACSL4. n = 5. **D, E** Erastin (20 μM) treatment up-regulates the expression of c-Myc and ACSL4 on both protein level and RNA level in a time dependent manner in MDA-MB-231 cells. Western blot images show the expression of indicated protein. RT-qPCR analyzes the expression of c-Myc and ACSL4. **F** c-Myc inhibitors increase the mRNA level of GOT1 in B16-F10 cells. RT-qPCR analyzes the expression of GOT1. 10074G5: 50 μM, n =3. **G** Overexpression of c-Myc suppresses the mRNA level of GOT1 in B16-F10 cells. RT-qPCR analyzes the expression of GOT1. **H** c-Myc inhibitors increase the protein level of NRF2 in HT1080 cells. Cells treated with 50 μM 10074-G5 for 24 h. n = 3. All data are mean ± SD. from n = 3 biological replicates. **P* < 0.05, ***P* < 0.01, ****P* < 0.001 by two-tailed *t*-test.


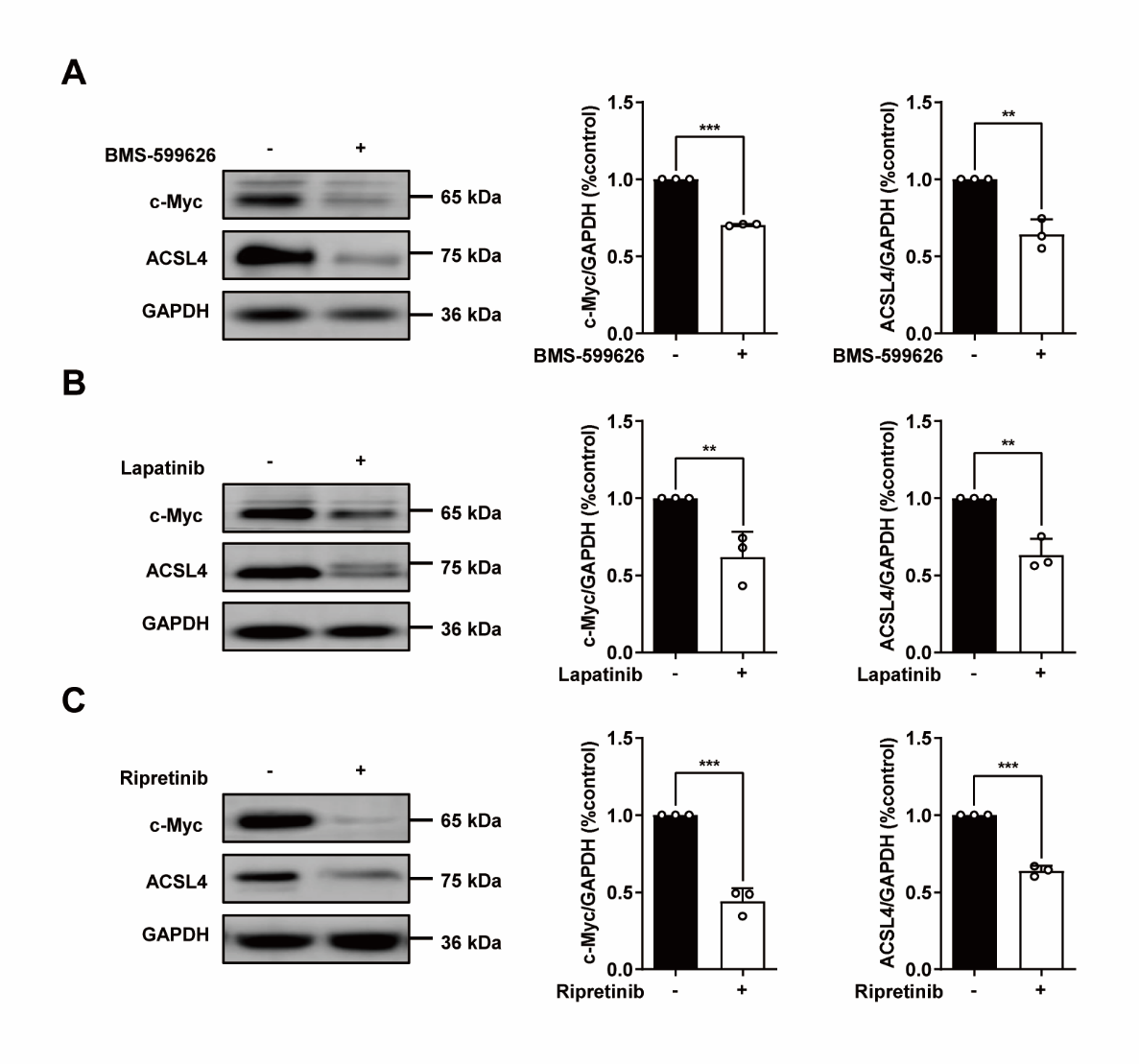


**Supplementary figure 4. RTKs promote ferroptosis by up-regulating c-Myc and ACSL4.**

**Fig.S4 A-C** RTKs inhibitors suppress the expression of c-Myc and ACSL4 in B16-F10 cells. Western blot images show the expression of indicated protein. BMS-599626: 20 μM, Lapatinib: 20 μM and Ripretinib: 20 μM. All data are mean ± SD. from n = 3 biological replicates. **P* <0.05, ***P* < 0.01, ****P* < 0.001 by two-tailed *t*-test.


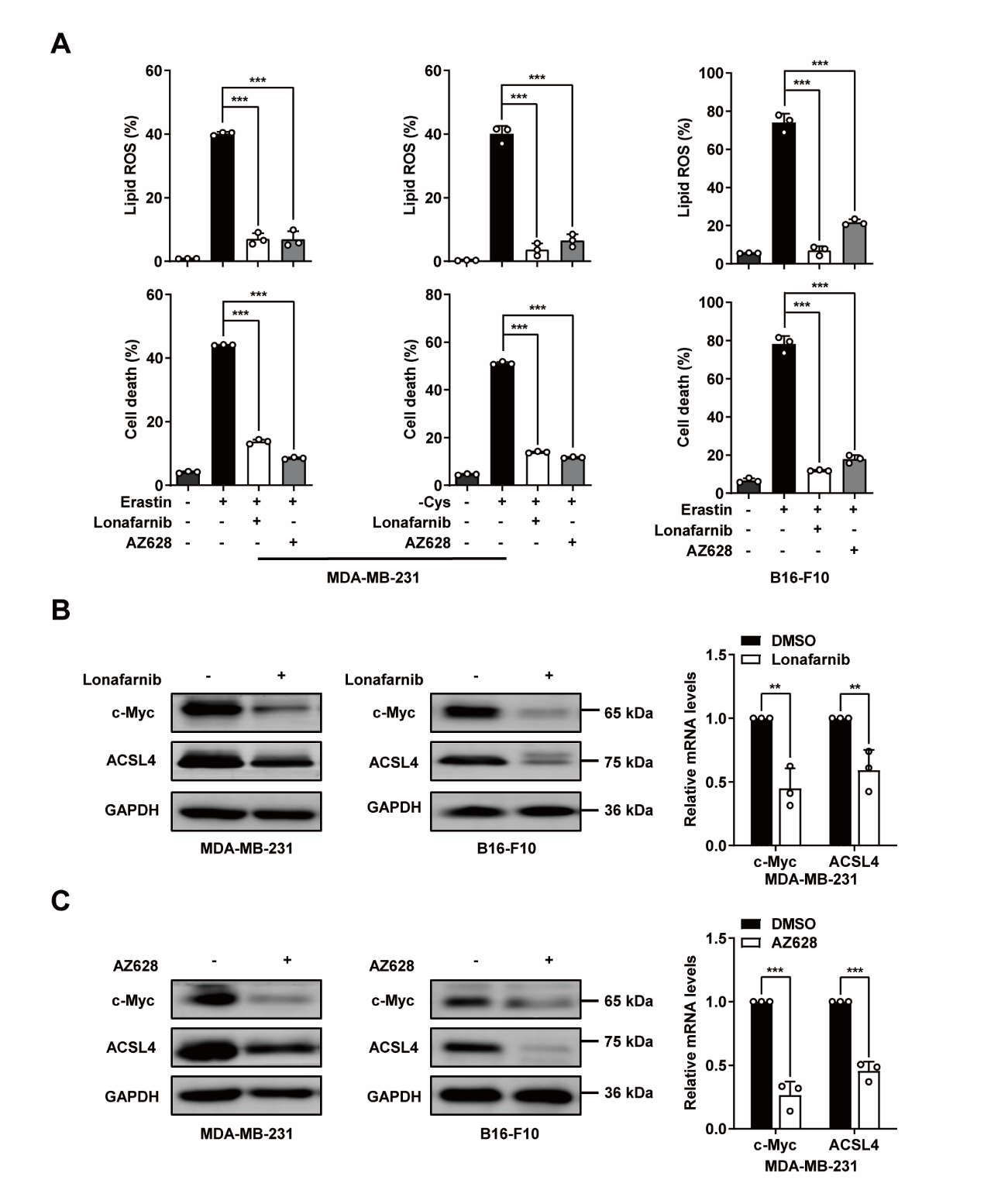


**Supplementary figure 5.** **RAS/RAF enhances the sensitivity of cancer cells to ferroptosis by up-regulating c-Myc and ACSL4.**

**Fig.S5 A** RAS inhibitor lonafarnib (10 μM) and RAF inhibitor AZ628 (10 μM) block ferroptosis in MDA-MB-231 and B16-F10 cells. MDA-MB-231 cells treated with erastin (10 μM for 18 h) or cysteine depleted medium (24 h) or B16-F10 cells treated with erastin (20 μM for 24 h) in the present of RAS inhibitor or RAF inhibitor or not to measure cell death. MDA-MB-231 cells treated with erastin (10 μM for 16 h) or cysteine depleted medium (22 h) or B16-F10 cells treated with erastin (20 μM for 22 h) in the presence of RAS inhibitor or RAF inhibitor or not to determine lipid ROS. **B** RAS inhibitor lonafarnib (10 μM) decreases the expression of cMyc and ACSL4 on both protein level and RNA level in the cells as indicated. Western blot images show the expression of indicated protein. RT-qPCR analyzes the expression of c-Myc and ACSL4 in MDA-MB-231 cells. **C** RAF inhibitor AZ628 (10 μM) decreases the expression of cMyc and ACSL4 on both protein level and RNA level in the cells as indicated. Western blot images show the expression of indicated protein. RT-qPCR analyzes the expression of c-Myc and ACSL4 in MDA-MB-231 cells. All data are mean ± SD. from n = 3 biological replicates. **P* < 0.05, ***P* < 0.01, ****P* < 0.001 by two-tailed *t*-test.
