# Supplementary material for: Oncogenic RTKs sensitize cancer cells to ferroptosis via c-Myc mediated upregulation of ACSL4
Source: Cell Death Dis. 2024 Nov 27;15(11):861. doi: 10.1038/s41419-024-07254-9 (PMC11603294; doi:10.1038/s41419-024-07254-9)

Figure1

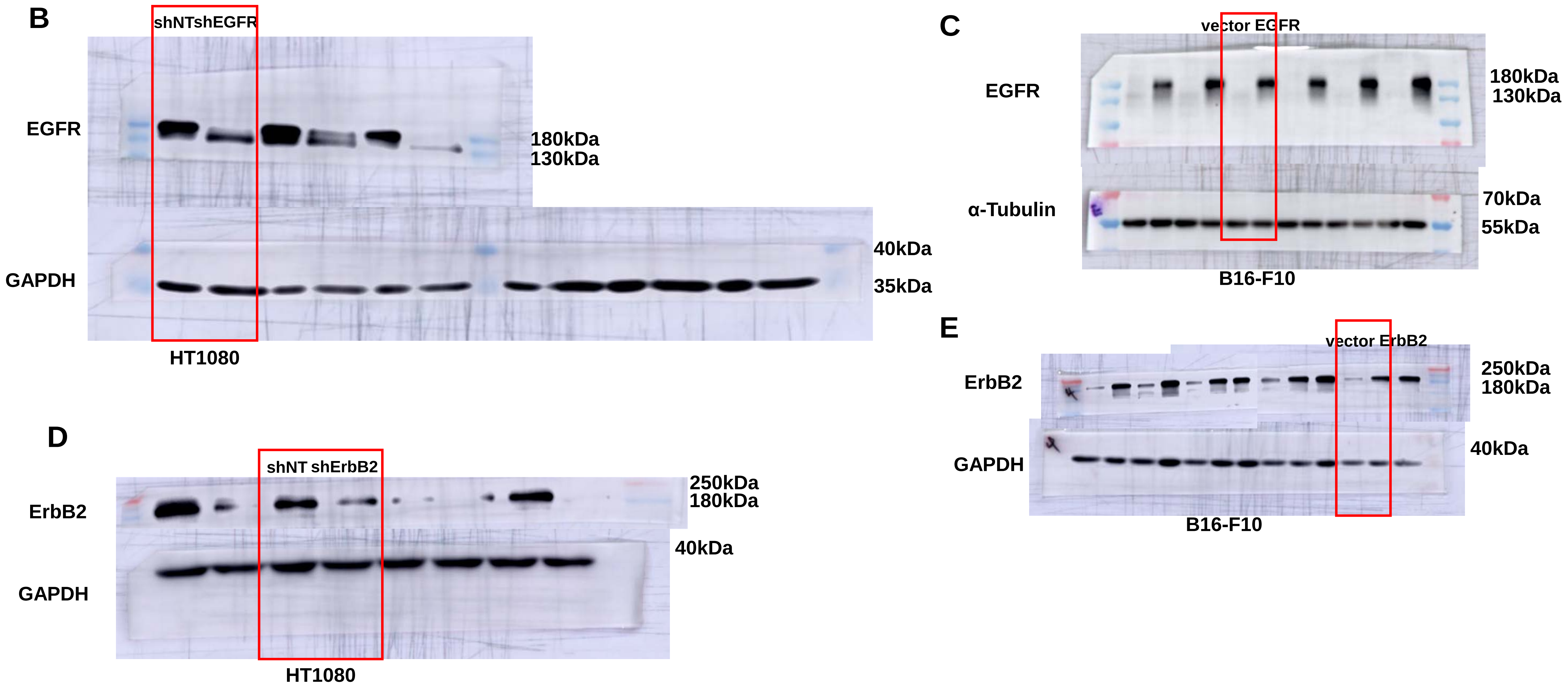
B
shNTshEGFR
EGFR
180kDa
130kDa
GAPDH
40kDa
35kDa
HT1080
C
vector EGFR
EGFR
180kDa
130kDa
α-Tubulin
70kDa
55kDa
B16-F10
D
shNT shErbB2
ErbB2
250kDa
180kDa
GAPDH
40kDa
HT1080
E
vector ErbB2
ErbB2
250kDa
180kDa
GAPDH
40kDa
B16-F10

Figure1

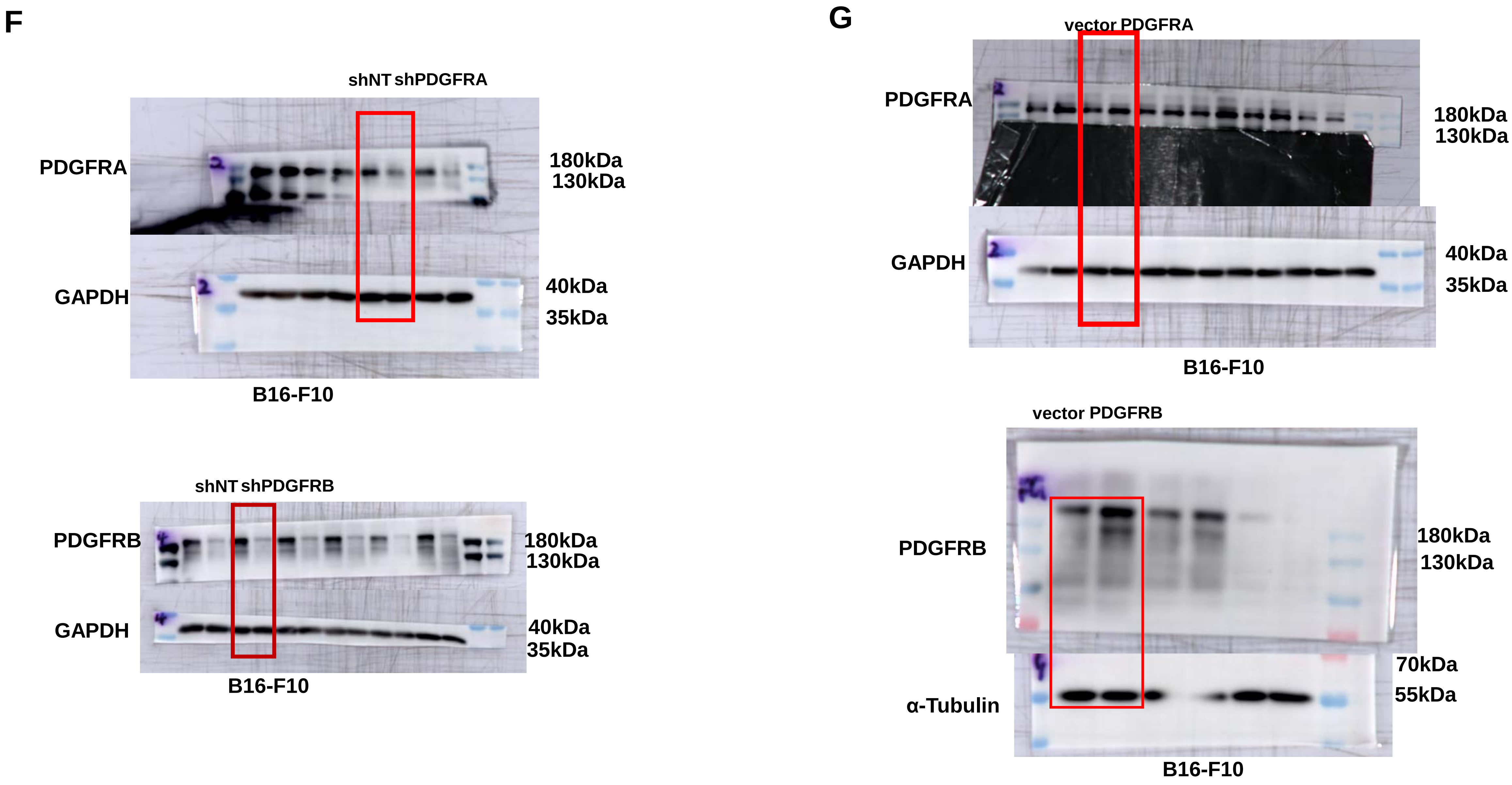
F
shNT shPDGFRA
PDGFRA
GAPDH
180kDa
130kDa
40kDa
35kDa
B16-F10
shNT shPDGFRB
PDGFRB
GAPDH
180kDa
130kDa
40kDa
35kDa
B16-F10
G
vector PDGFRA
PDGFRA
GAPDH
180kDa
130kDa
40kDa
35kDa
B16-F10
vector PDGFRB
PDGFRB
α-Tubulin
180kDa
130kDa
70kDa
55kDa
B16-F10

Figure2

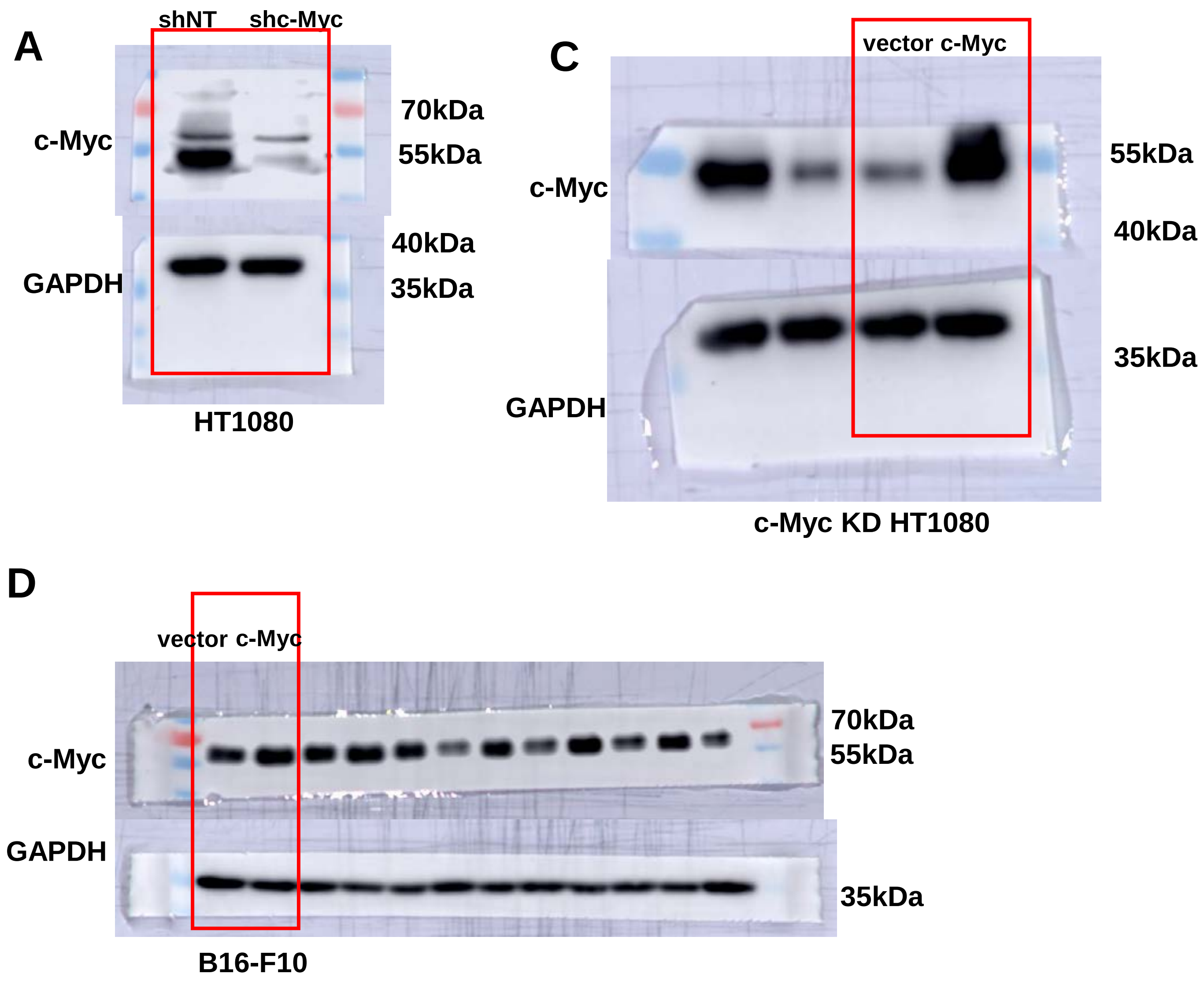
A
shNT
shc-Myc
c-Myc
GAPDH
70kDa
55kDa
40kDa
35kDa
HT1080
C
vector c-Myc
c-Myc
GAPDH
55kDa
40kDa
35kDa
c-Myc KD HT1080
D
vector c-Myc
c-Myc
GAPDH
70kDa
55kDa
35kDa
B16-F10

Figure3

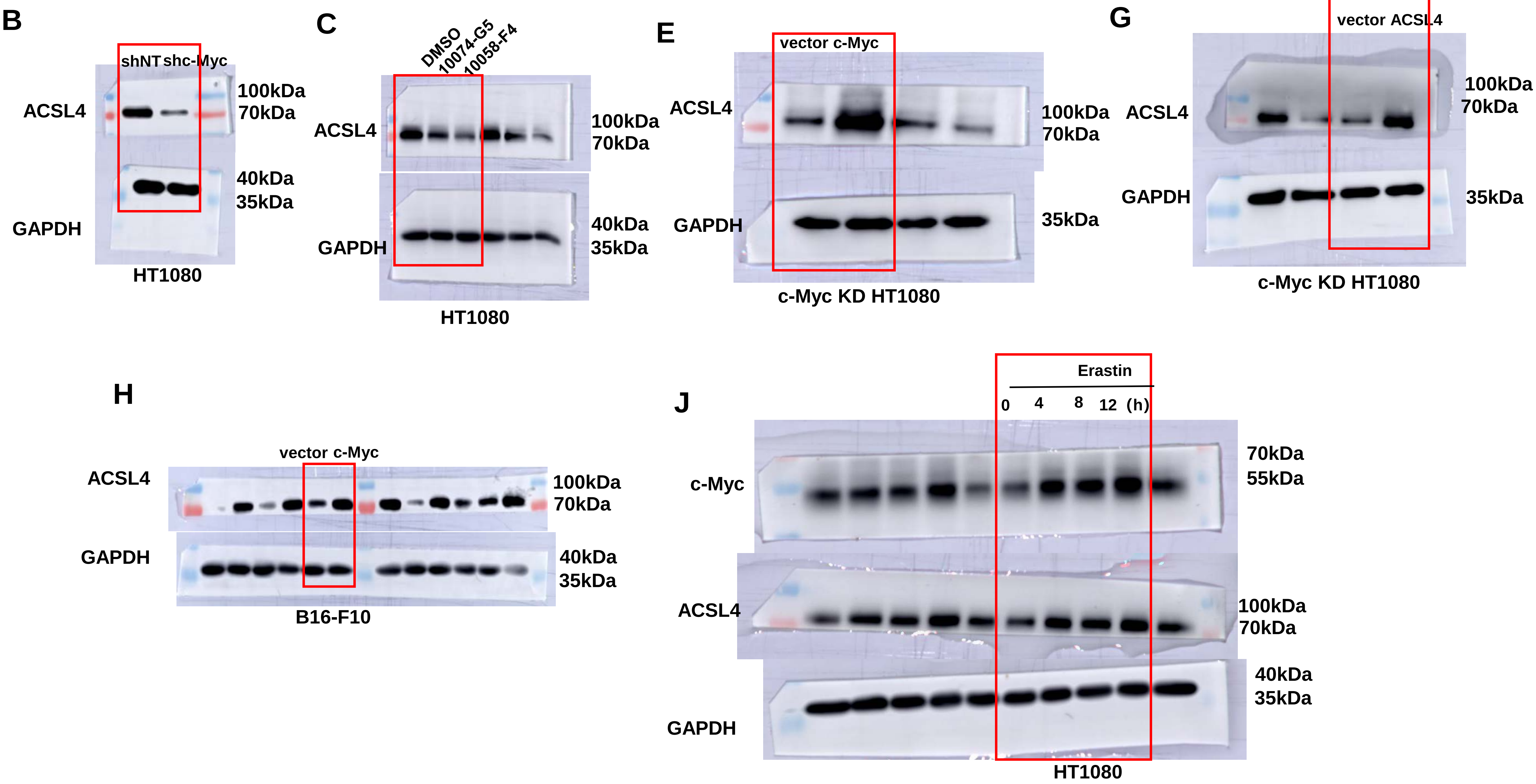
B
shNT shc-Myc
ACSL4
GAPDH
100kDa
70kDa
40kDa
35kDa
HT1080
C
DMSO
10074-G5
10058-F4
ACSL4
GAPDH
100kDa
70kDa
40kDa
35kDa
HT1080
E
vector c-Myc
ACSL4
GAPDH
100kDa
70kDa
35kDa
c-Myc KD HT1080
G
vector ACSL4
ACSL4
GAPDH
100kDa
70kDa
35kDa
c-Myc KD HT1080
H
vector c-Myc
ACSL4
GAPDH
100kDa
70kDa
40kDa
35kDa
B16-F10
J
Erastin
0 4 8 12 (h)
c-Myc
ACSL4
GAPDH
70kDa
55kDa
100kDa
70kDa
40kDa
35kDa
HT1080

Figure4

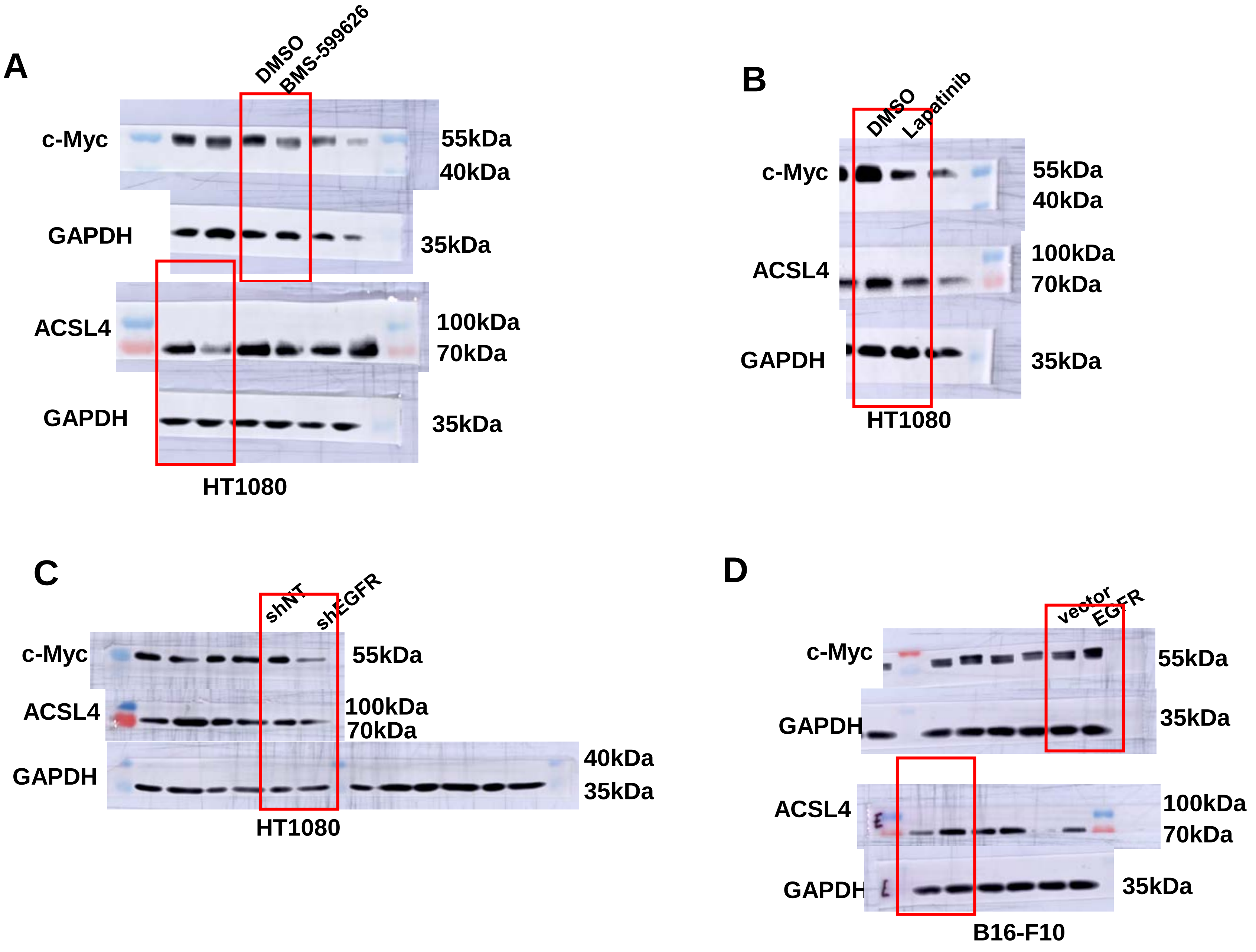

A
DMSO
BMS-599626
c-Myc
55kDa
40kDa
GAPDH
35kDa
ACSL4
100kDa
70kDa
GAPDH
35kDa
HT1080
B
DMSO
Lapatinib
c-Myc
55kDa
40kDa
ACSL4
100kDa
70kDa
GAPDH
35kDa
HT1080
C
shNT
shEGFR
c-Myc
55kDa
ACSL4
100kDa
70kDa
GAPDH
40kDa
35kDa
HT1080
D
vector
EGFR
c-Myc
55kDa
GAPDH
35kDa
ACSL4
100kDa
70kDa
GAPDH
35kDa
B16-F10

Figure4

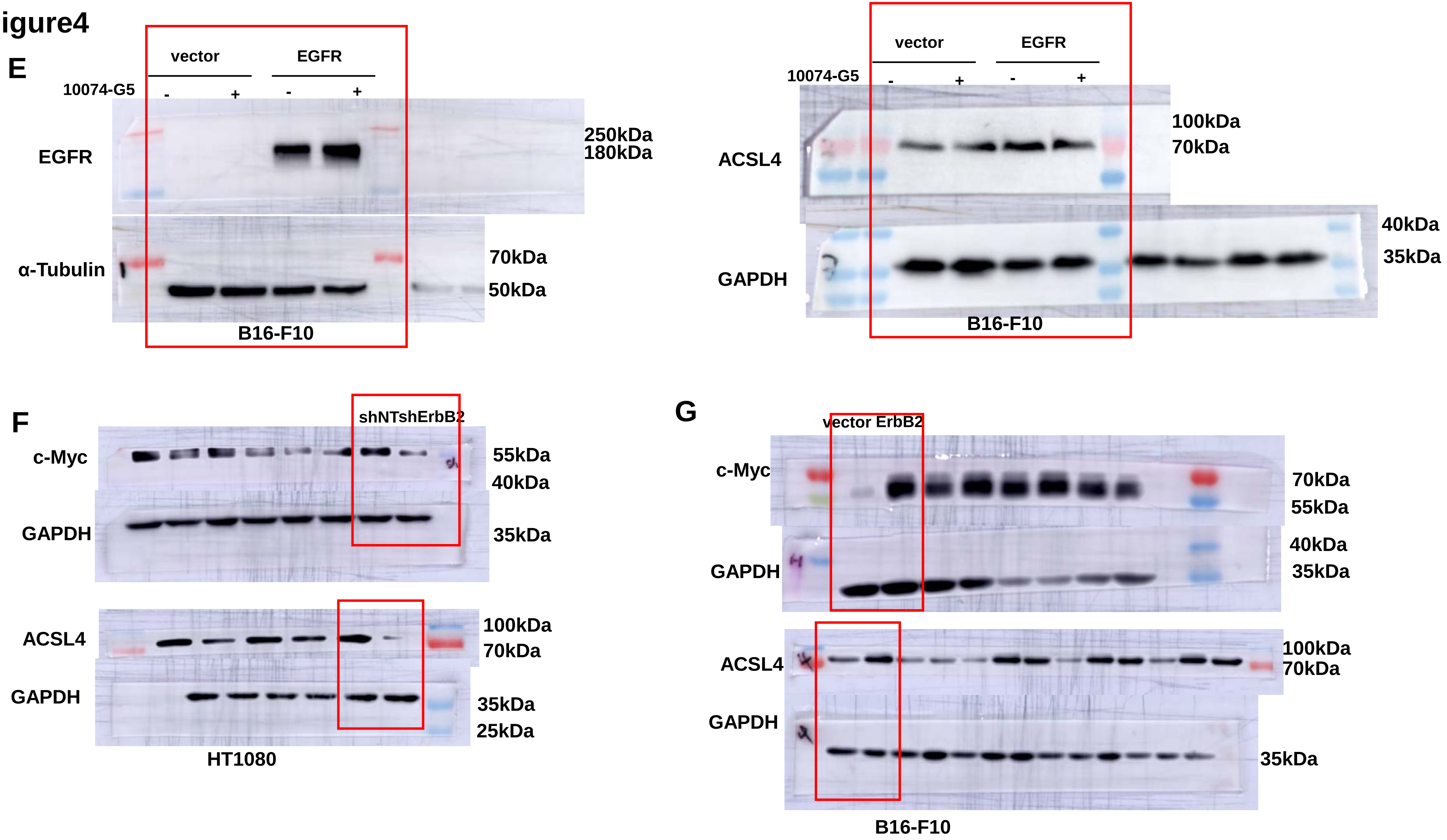
E
vector
EGFR
10074-G5
-
+
-
+
EGFR
250kDa
180kDa
α-Tubulin
70kDa
50kDa
B16-F10
vector
EGFR
10074-G5
-
+
-
+
ACSL4
100kDa
70kDa
GAPDH
40kDa
35kDa
B16-F10
F
shNTshErbB2
c-Myc
55kDa
40kDa
GAPDH
35kDa
ACSL4
100kDa
70kDa
GAPDH
35kDa
25kDa
HT1080
G
vector ErbB2
c-Myc
70kDa
55kDa
GAPDH
40kDa
35kDa
ACSL4
100kDa
70kDa
GAPDH
35kDa
B16-F10

Figure4

H

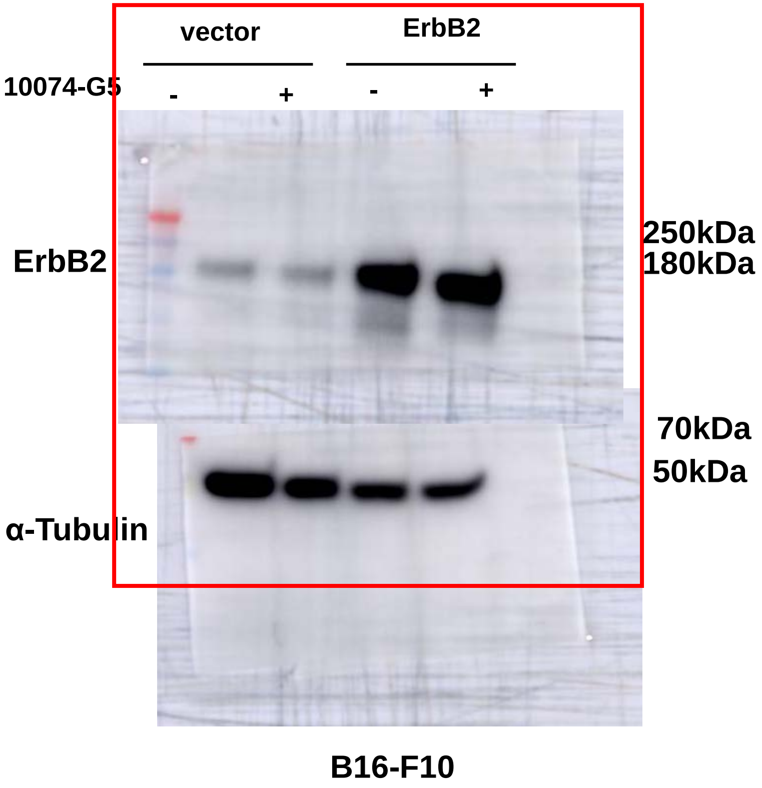
vector
ErbB2
10074-G5
-
+
-
+
ErbB2
α-Tubulin
250kDa
180kDa
70kDa
50kDa
B16-F10

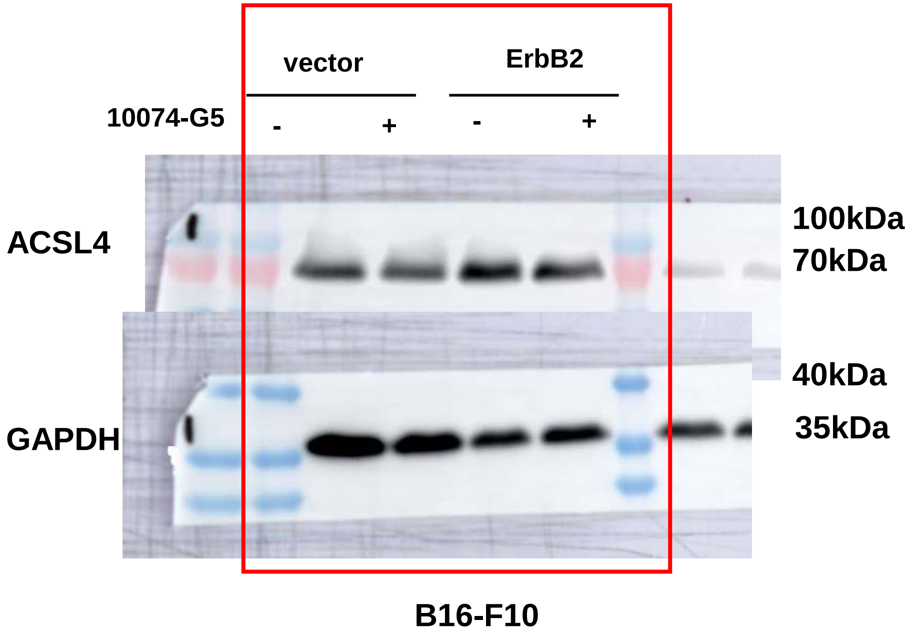
vector
ErbB2
10074-G5
-
+
-
+
ACSL4
GAPDH
100kDa
70kDa
40kDa
35kDa
B16-F10

Figure4

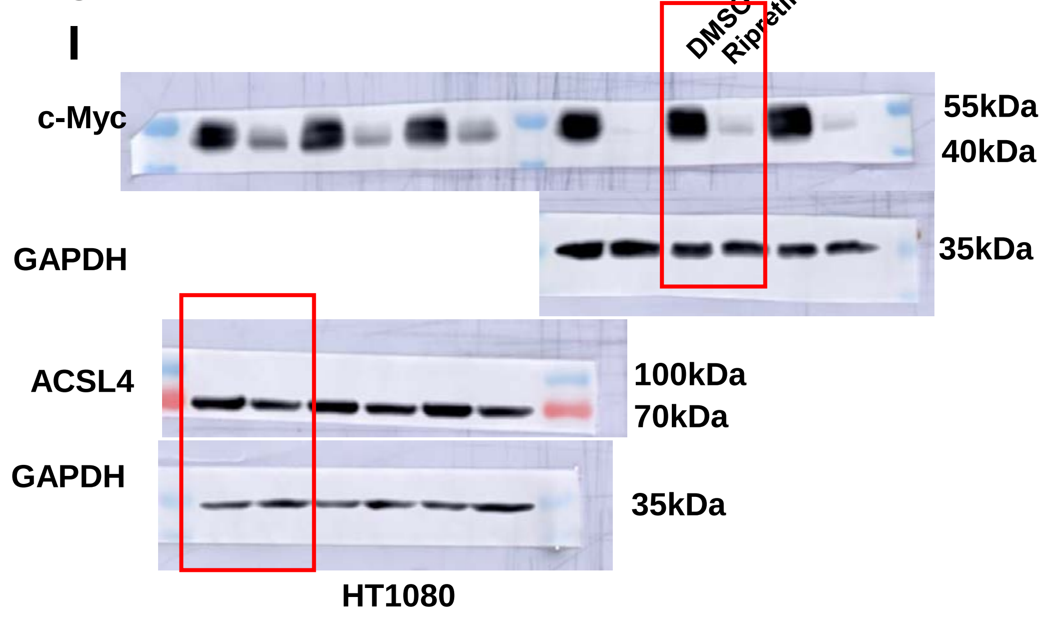

I
DMSO
Ripretinib
c-Myc
55kDa
40kDa
GAPDH
35kDa
ACSL4
100kDa
70kDa
GAPDH
35kDa
HT1080

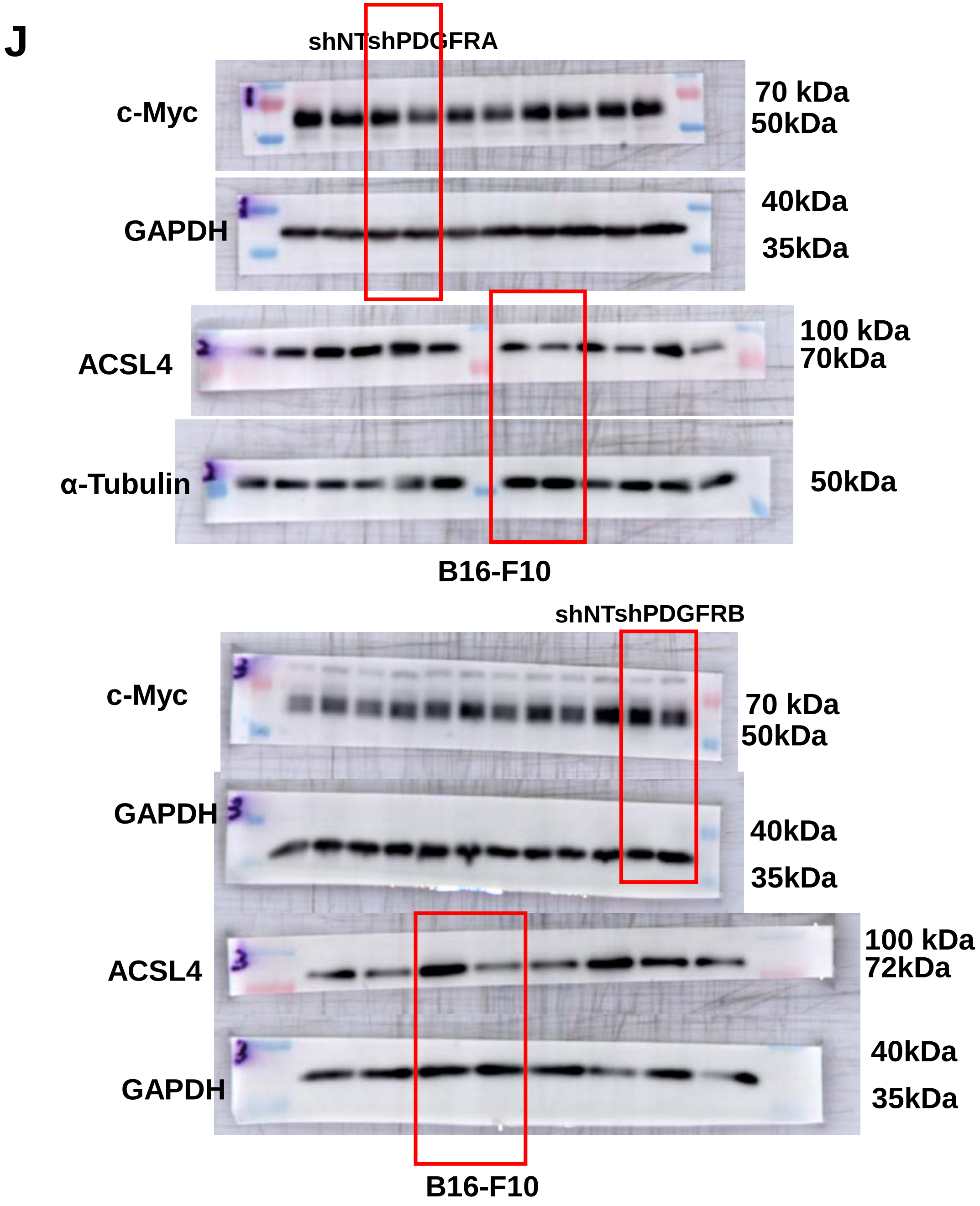

J
shNT shPDGFRA
c-Myc
70 kDa
50kDa
GAPDH
40kDa
35kDa
ACSL4
100 kDa
70kDa
α-Tubulin
50kDa
B16-F10
shNT shPDGFRB
c-Myc
70 kDa
50kDa
GAPDH
40kDa
35kDa
ACSL4
100 kDa
72kDa
GAPDH
40kDa
35kDa
B16-F10

Figure4

K

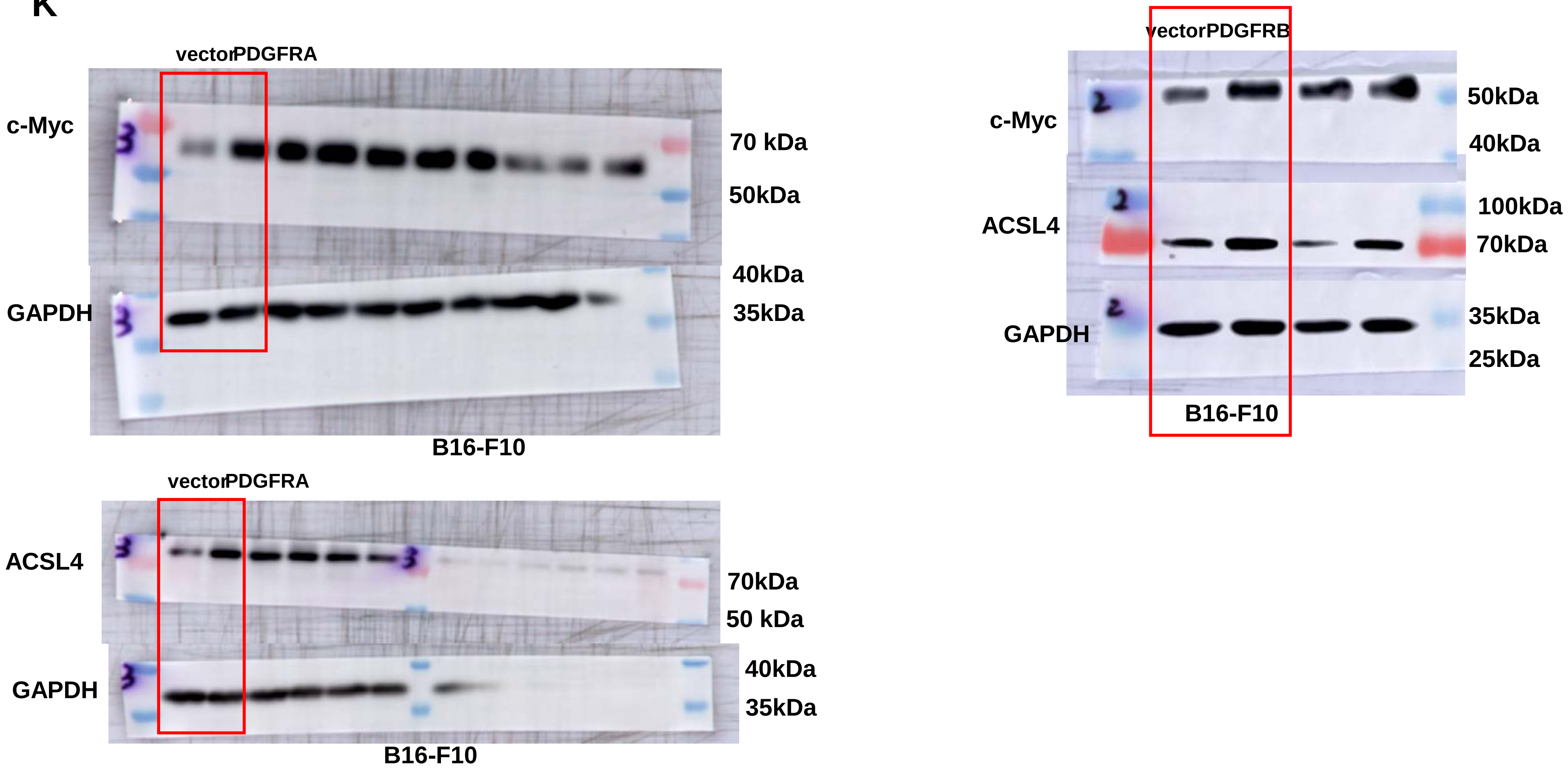

vectorPDGFRA
c-Myc
70 kDa
50kDa
40kDa
35kDa
GAPDH
B16-F10
vectorPDGFRA
ACSL4
70kDa
50 kDa
40kDa
35kDa
GAPDH
B16-F10
vectorPDGFRB
c-Myc
50kDa
40kDa
ACSL4
100kDa
70kDa
GAPDH
35kDa
25kDa
B16-F10

Figure4

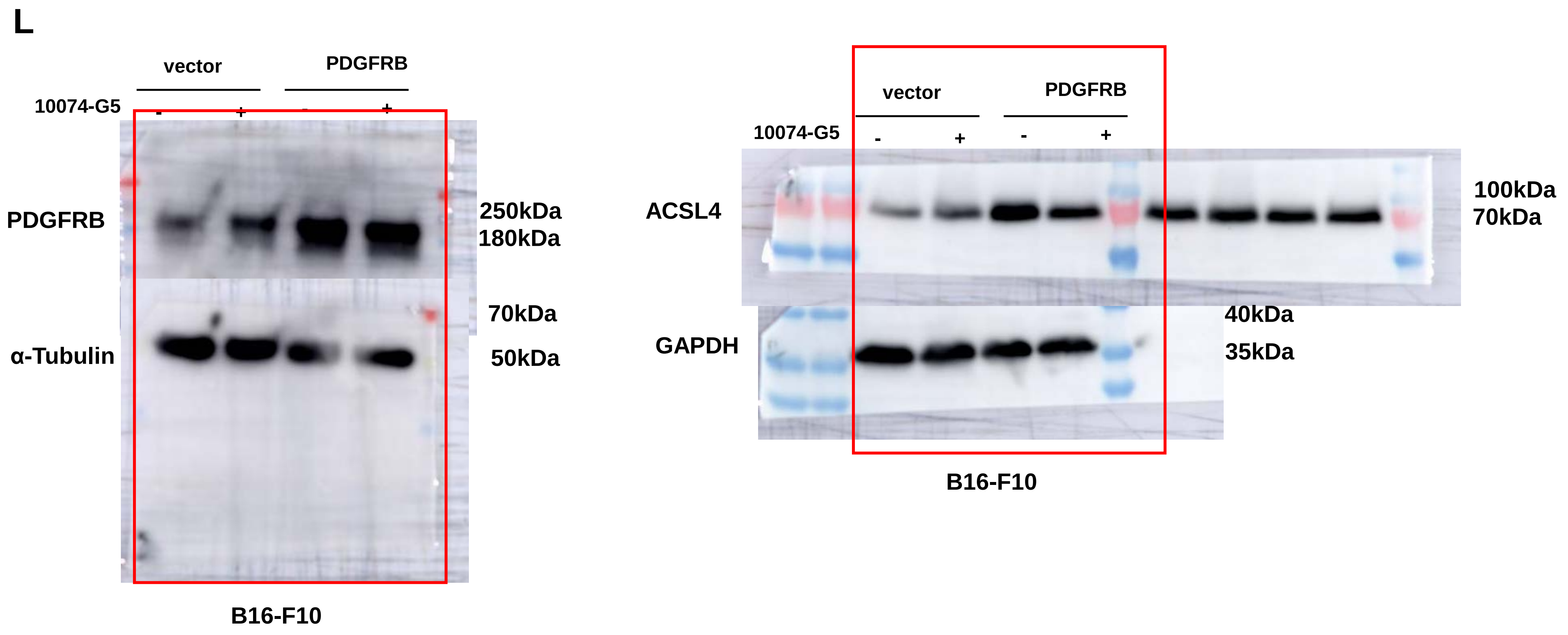

L
vector
PDGFRB
10074-G5
-
+
-
+
PDGFRB
α-Tubulin
250kDa
180kDa
70kDa
50kDa
B16-F10
vector
PDGFRB
10074-G5
-
+
-
+
ACSL4
GAPDH
100kDa
70kDa
40kDa
35kDa
B16-F10

Figure5

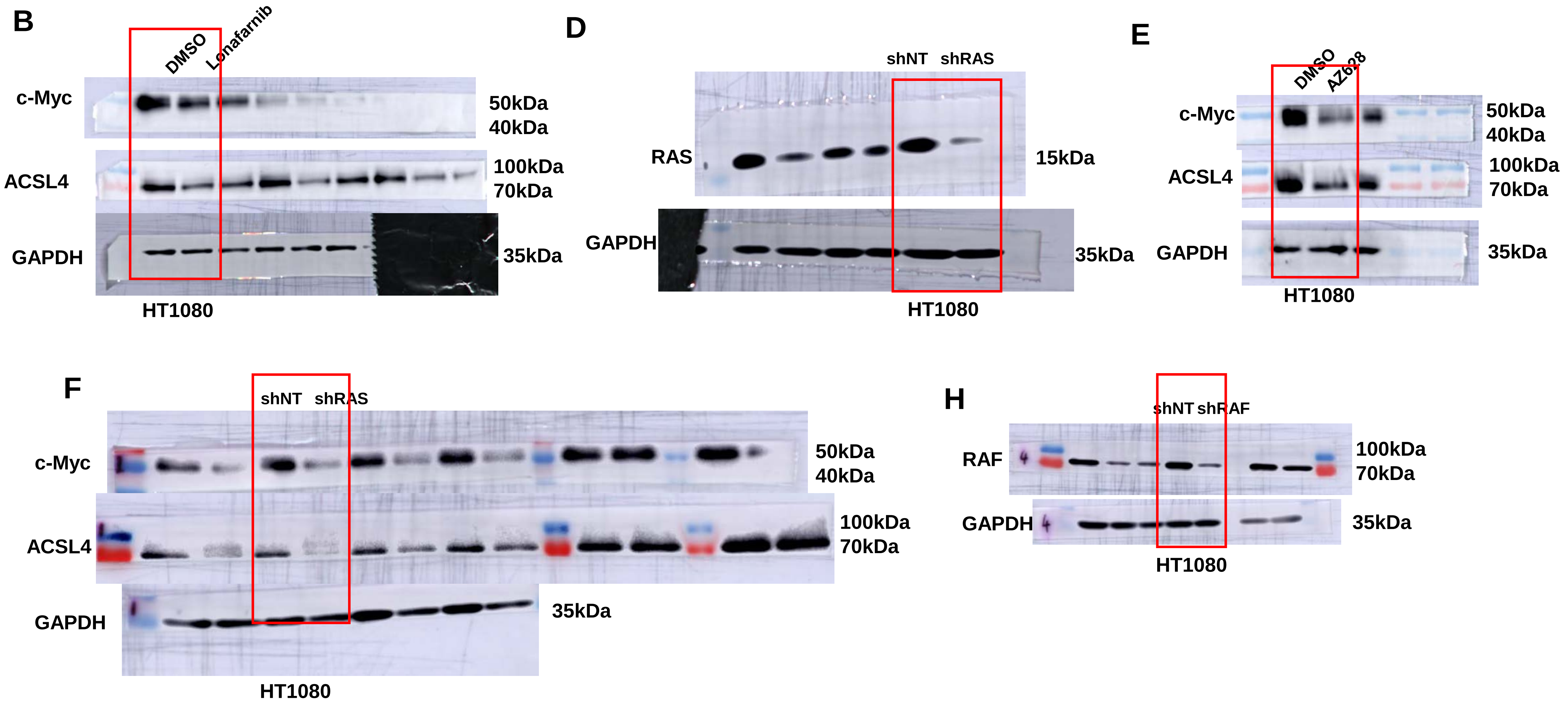
B
DMSO
Lonafarnib
c-Myc
50kDa
40kDa
ACSL4
100kDa
70kDa
GAPDH
35kDa
HT1080
D
shNT
shRAS
RAS
15kDa
GAPDH
35kDa
HT1080
E
DMSO
AZ628
c-Myc
50kDa
40kDa
ACSL4
100kDa
70kDa
GAPDH
35kDa
HT1080
F
shNT
shRAS
c-Myc
50kDa
40kDa
ACSL4
100kDa
70kDa
GAPDH
35kDa
HT1080
H
shNT
shRAF
RAF
100kDa
70kDa
GAPDH
35kDa
HT1080

Figure5

I

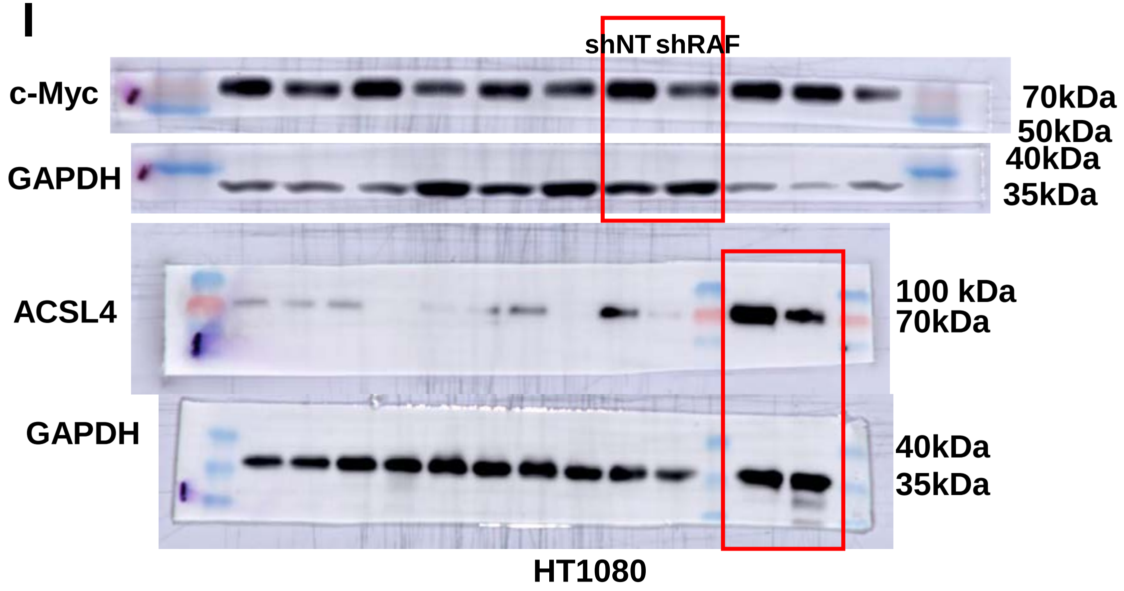

Supplementary figure2 (related to Figure 2).

C

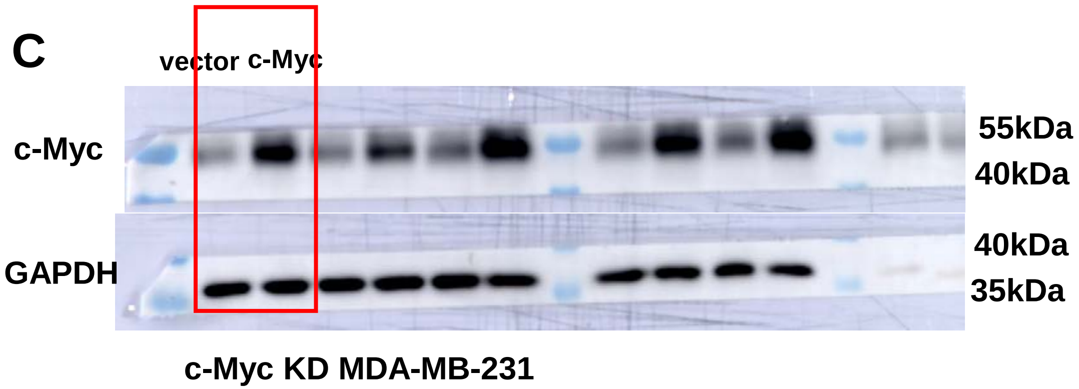

Supplementary figure2 (related to Figure 2).

A

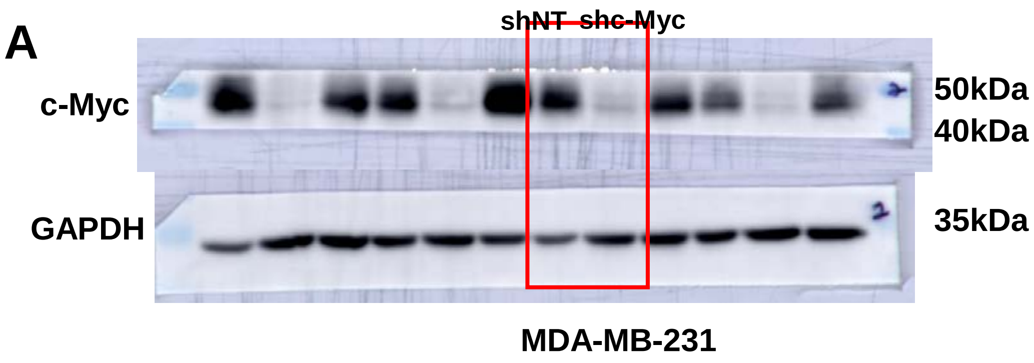

Supplementary figure3 (related to Figure 3).

A

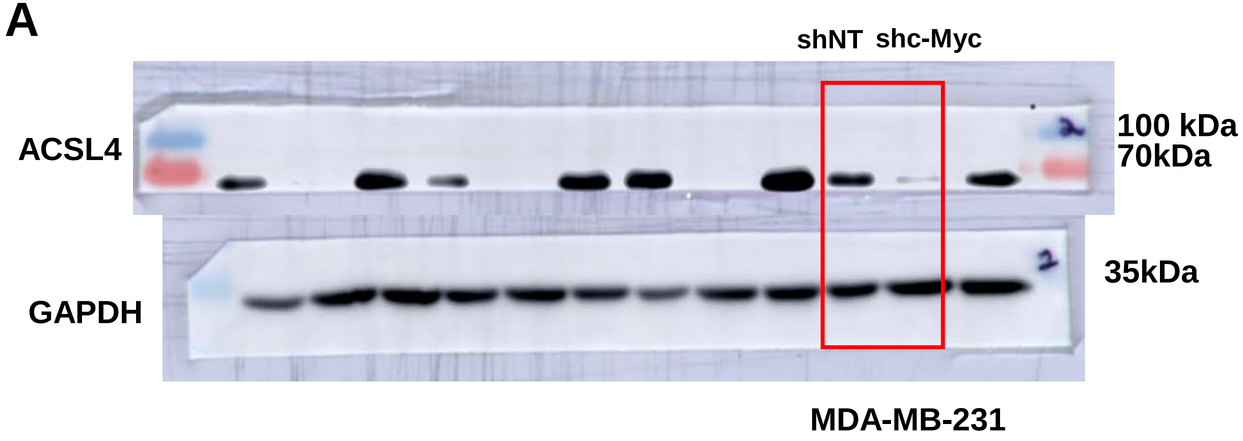

Supplementary figure3 (related to Figure 3).

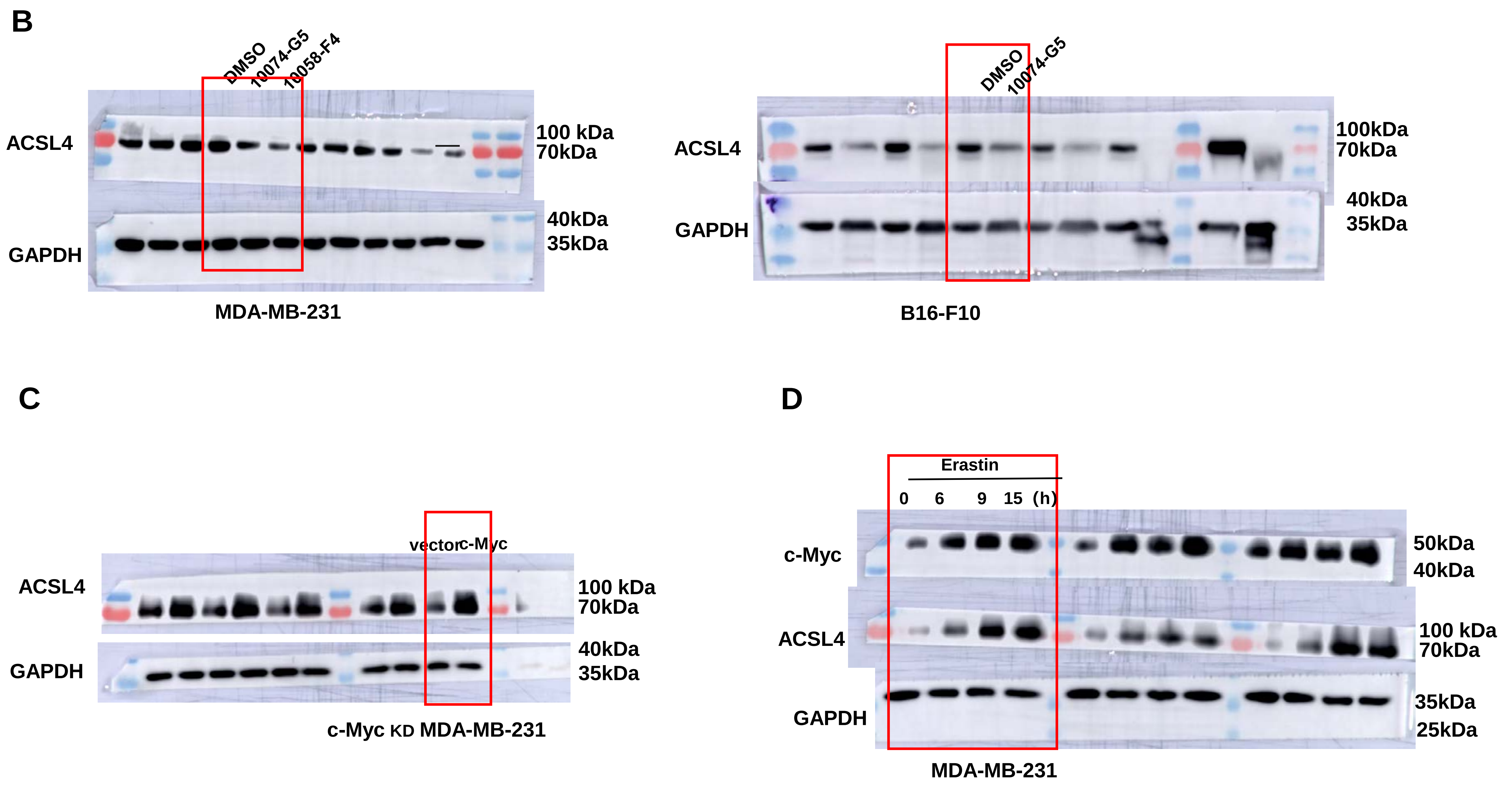

Supplementary figure3 (related to Figure 3).

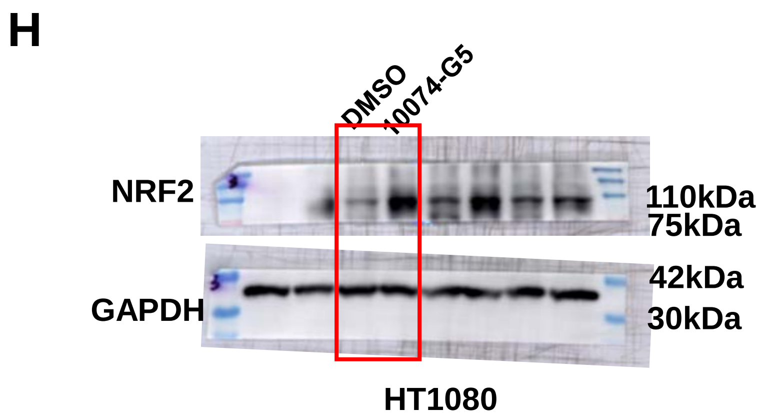

Supplementary figure4 (related to Figure 4).

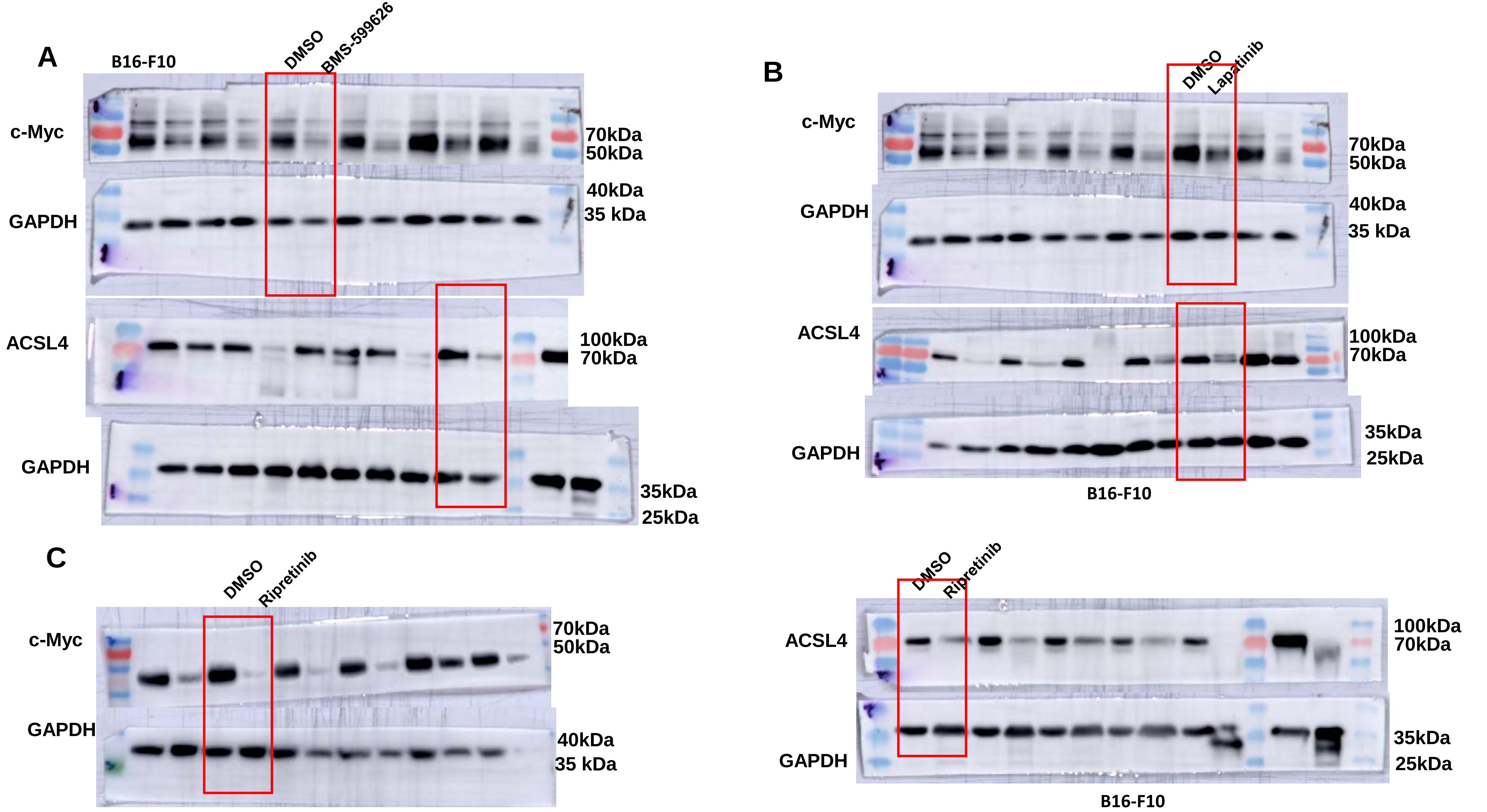

Supplementary figure5 (related to Figure 5).

B

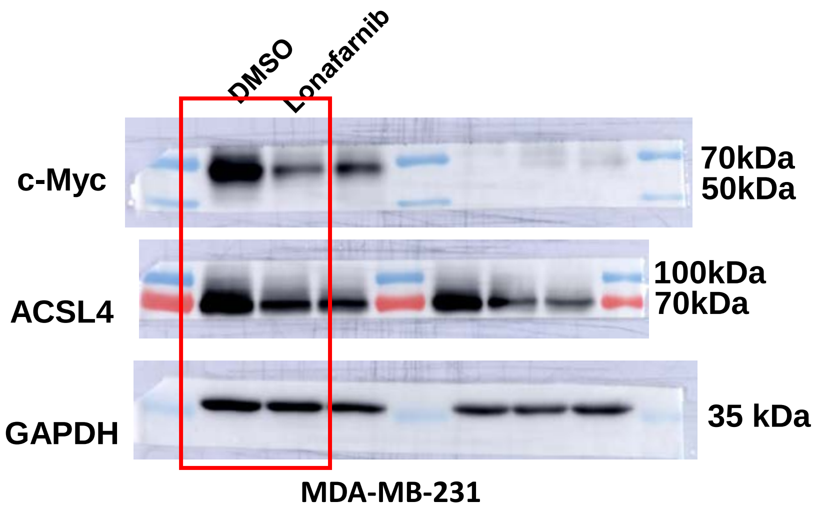

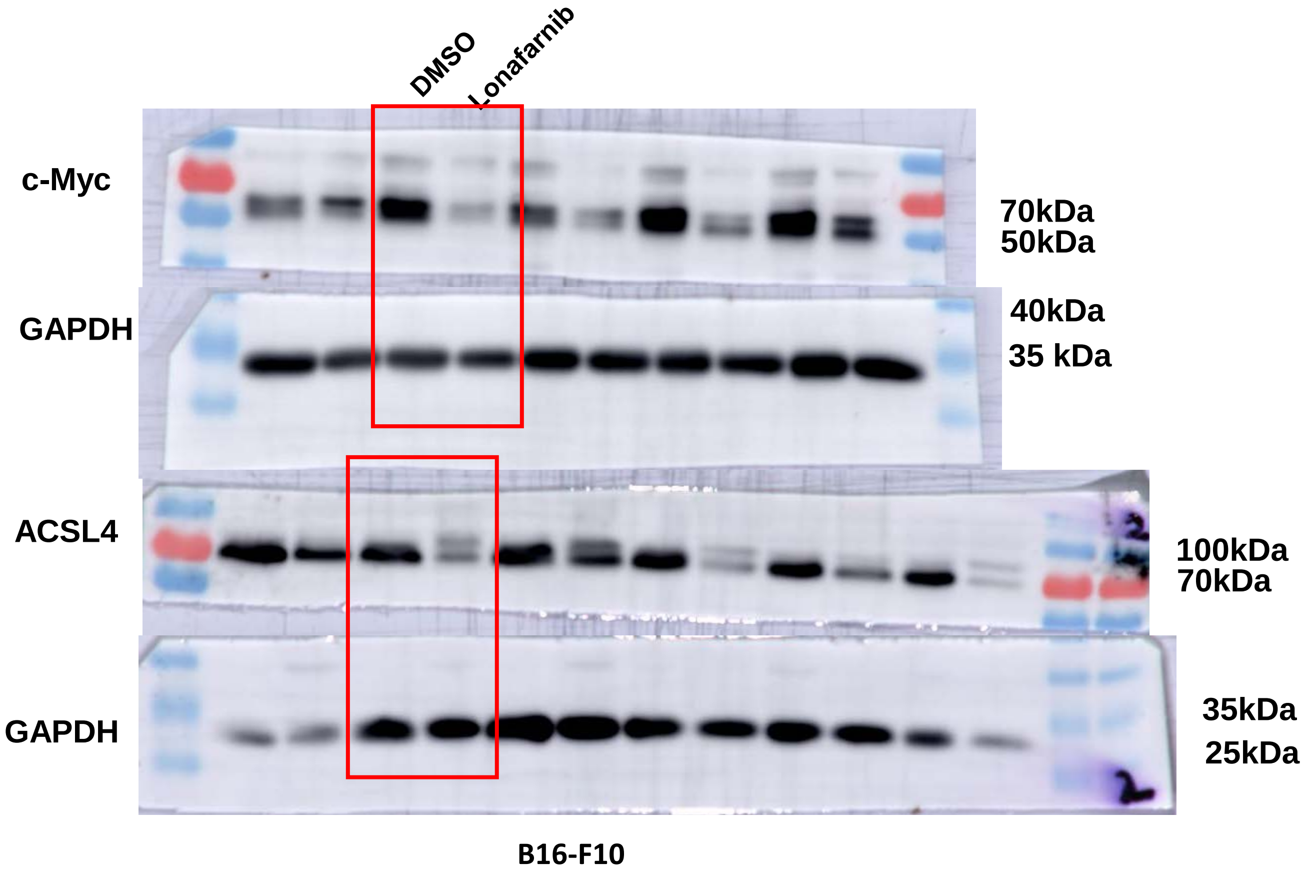

Supplementary figure5 (related to Figure 5).

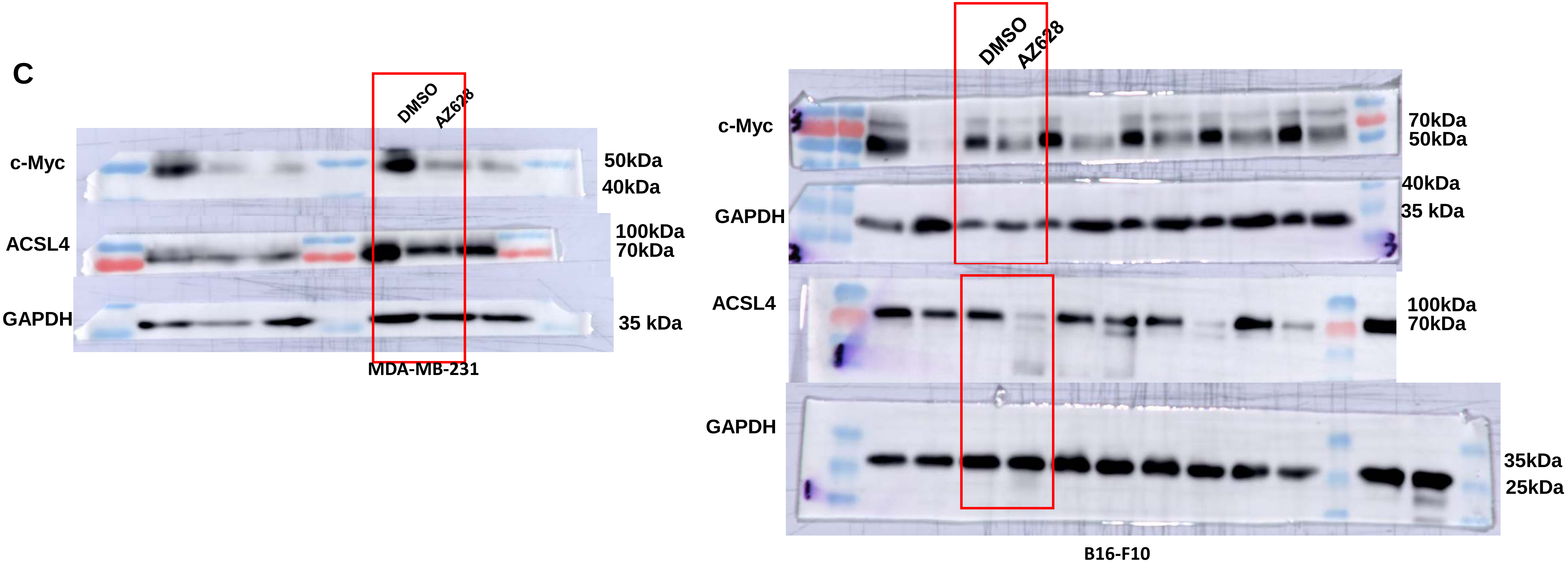

Supplement: Supplementary file 3 — Original full length Western blots [file 41419_2024_7254_MOESM3_ESM.pdf]
